# The Etiology of Pneumonia in HIV-uninfected Children in Kilifi, Kenya: Findings From the Pneumonia Etiology Research for Child Health (PERCH) Study

**DOI:** 10.1097/INF.0000000000002653

**Published:** 2021-08-25

**Authors:** Juliet O. Awori, Alice Kamau, Susan Morpeth, Sidi Kazungu, Micah Silaba, Joyce Sande, Angela Karani, Sammy Nyongesa, Salim Mwarumba, Robert Musyimi, Anne Bett, Siti Wande, Mohammed Shebe, Mwanajuma Ngama, Patrick K. Munywoki, Neema Muturi, D. James Nokes, Daniel R. Feikin, David R. Murdoch, Christine Prosperi, Katherine L. O’Brien, Maria Deloria Knoll, Laura L. Hammitt, J. Anthony G. Scott

**Affiliations:** From the * Epidemiology and Demography Department, KEMRI-Wellcome Trust Research Programme, CGMR-Coast, Kilifi, Kenya; † Department of Infectious Disease Epidemiology, London School of Hygiene & Tropical Medicine, London, United Kingdom; ‡ Department of Radiology, Aga Khan University Hospital, Nairobi, Kenya; § Clinical Sciences Department, KEMRI-Wellcome Trust Research Programme, CGMR-Coast, Kilifi, Kenya; ¶ School of Life Sciences and WIDER, University of Warwick, Coventry, United Kingdom; ‖ Department of International Health, International Vaccine Access Center, Johns Hopkins Bloomberg School of Public Health, Baltimore, Maryland; ** Department of Pathology, University of Otago, Christchurch, New Zealand; ††† Microbiology Unit, Canterbury Health Laboratories, Christchurch, New Zealand; ‡‡‡ Nuffield Department of Tropical Medicine, Oxford University, Oxford, United Kingdom.

**Keywords:** Kenya, pneumonia, etiology, childhood, Pneumonia Etiology Research for Child Health

## Abstract

In the 1980s, *Streptococcus pneumoniae* and *Haemophilus influenzae* were identified as the principal causes of severe pneumonia in children. We investigated the etiology of severe childhood pneumonia in Kenya after introduction of conjugate vaccines against *H. influenzae* type b, in 2001, and *S. pneumoniae*, in 2011.

**Methods:**

We conducted a case–control study between August 2011 and November 2013 among residents of the Kilifi Health and Demographic Surveillance System 28 days to 59 months of age. Cases were hospitalized at Kilifi County Hospital with severe or very severe pneumonia according to the 2005 World Health Organization definition. Controls were randomly selected from the community and frequency matched to cases on age and season. We tested nasal and oropharyngeal samples, sputum, pleural fluid, and blood specimens and used the Pneumonia Etiology Research for Child Health Integrated Analysis, combining latent class analysis and Bayesian methods, to attribute etiology.

**Results:**

We enrolled 630 and 863 HIV-uninfected cases and controls, respectively. Among the cases, 282 (44%) had abnormal chest radiographs (CXR positive), 33 (5%) died in hospital, and 177 (28%) had diagnoses other than pneumonia at discharge. Among CXR-positive pneumonia cases, viruses and bacteria accounted for 77% (95% CrI: 67%–85%) and 16% (95% CrI: 10%–26%) of pneumonia attribution, respectively. Respiratory syncytial virus, *S. pneumoniae* and *H. influenza*, accounted for 37% (95% CrI: 31%–44%), 5% (95% CrI: 3%–9%), and 6% (95% CrI: 2%–11%), respectively.

**Conclusions:**

Respiratory syncytial virus was the main cause of CXR-positive pneumonia. The small contribution of *H. influenzae* type b and pneumococcus to pneumonia may reflect the impact of vaccine introductions in this population.

## Abstract 2

Supplemental Digital Content is available in the text.

Pneumonia is the leading cause of death in children <5 years of age in developing countries.^1^ *Streptococcus pneumoniae* and *Haemophilus influenzae* were the main causes of severe pneumonia and the principal pathogens against which treatment is targeted in the Integrated Management of Childhood illness guidelines.^2^

*H. influenzae* type b (Hib) vaccine and the 10-valent pneumococcal conjugate vaccine (PCV10) were introduced into the Kenya National Immunization Program in November 2001 and January 2011, respectively. In Kilifi, invasive disease caused by Hib declined by 88% in the 3 years after the vaccine was introduced^3^ and this was sustained for a further 9 years.^4^ PCV10 was introduced in Kilifi with a catch-up campaign for children 12–59 months of age to accelerate herd protection.^5^ At the end of 2011, vaccine coverage in children 2–11 months (≥2 doses) and 12–59 months (≥1 dose) was 80% and 66%, respectively.^6^ By 2016, the incidence of invasive pneumococcal disease caused by vaccine serotypes declined by 92% among children <5 years of age^6^; the incidence of radiologically confirmed pneumonia and clinically defined pneumonia declined by 48% and 27%, respectively.^7^

Given the success of these vaccines across the world,^8–10^ the etiologic profile of childhood pneumonia is expected to change following their introduction. The remaining pathogens are likely to be more diverse, and current treatment strategies against childhood pneumonia are likely to be ineffective. We need to direct new drug and vaccine development against the remaining pathogens.^11,12^

The Pneumonia Etiology Research for Child Health (PERCH) study was a 7-country case–control study evaluating the etiology of pneumonia among hospitalized children in developing countries.^13^ In Kilifi, PERCH was nested within the Kilifi Health and Demographic Surveillance System (KHDSS), which has conducted long-term surveillance of invasive bacterial diseases and viral respiratory pathogens for 2 decades. Kilifi is typical of much of rural sub-Saharan Africa, with low HIV prevalence, a mature Hib vaccine program and a recent PCV program.

## STUDY POPULATION AND METHODS

### Study Site

KHDSS covers an area of 891 km^2^ around Kilifi County Hospital (KCH) located on the Kenya Coast and follows a population of approximately 270,000 for vital events and migration, through 4 monthly household visits. Hospitalization at KCH, including electronic clinical and laboratory records, is captured in the KHDSS database through real-time linkage of each patient to the KHDSS population register.^14^

Kilifi County has a predominantly rural population of subsistence farmers, 58% of whom live below the National poverty line.^15^ It has 2 rainy seasons: April to July and October to December.^16^ Malaria transmission peaks during the rainy seasons, but overall, malaria has been declining over the last 15 years.^17^ Under 5 and infant mortality ratios in the KHDSS were 34.6 and 23.4 per 1000 live births, respectively, between 2011 and 2013. Pneumonia and malaria were the principal causes of death among infants and children 1–4 years of age, respectively, between 2008 and 2011.^18^ Annual HIV prevalence estimates among women attending antenatal screening at KCH were 2.6%–3.2% between 2011 and 2013. The prevalence of wasting, underweight and stunting (given as Z scores of <−2 SD in weight-for-height, weight-for-age and height-for-age) were 4%, 17% and 39%, respectively, in children <5 years of age in Kilifi County.^19^

### Selection of Participants

All pediatric admissions to KCH from August 15, 2011, to November 15, 2013, were screened for eligibility. Clinical officers assessed most children at the outpatient clinic; very sick children, or those presenting out of hours, were assessed on the pediatric wards. Study clinicians examined all admissions and recorded clinical data directly onto a standardized computer record. Children 28 days to 59 months of age, resident within the KHDSS and admitted with clinical features of 2005 World Health Organization (WHO)-defined severe or very severe pneumonia, were eligible to participate.^20,21^ Eligibility was assessed in real time by a computer algorithm applying PERCH inclusion and exclusion criteria (Table, Supplemental Digital Content 1, http://links.lww.com/INF/D871).

Between December 7, 2012, and February 11, 2013, recruitment was interrupted by a nationwide nurses’ strike which closed the general pediatric ward. During this time, recruitment was restricted to very sick children in the High Dependency Unit. The study was extended by 3 months (August 16 to November 15, 2013) to compensate for this interruption.

Controls were selected randomly from the KHDSS population register. Children were eligible if they did not have WHO-defined severe or very severe pneumonia. They were frequency matched to cases by age and season but with a minimum monthly recruitment target of 25 controls. We visited a homestead ≥3 times before concluding that the selected control was unavailable and choosing a replacement from the random list. For controls, enrolment and sample collection was done at participants’ homes.

### Clinical Samples and Laboratory Analyses

At admission, blood samples were collected for culture, serology, microscopic examination for malaria parasites, and polymerase chain reaction (PCR) to detect *S. pneumoniae*. Rapid HIV antibody testing was done as part of routine care. Nasopharyngeal and oropharyngeal swab (NPOP) samples were collected within 8 hours of admission for PCR testing using a 33-pathogen multiplex quantitative PCR (FTD Resp-33; Fast-track Diagnostics, Sliema, Malta) and pneumococcal culture (NP swabs only). Induced sputum (IS) samples, for culture, *Mycobacterium tuberculosis* (MTB) and PCR testing, were collected within 24 hours of admission. In children with suspected MTB, a second IS sample was collected for additional MTB testing 48 hours after admission or a gastric aspirate sample where IS was clinically contraindicated. Pleural fluid samples were collected for culture and PCR in children where a pleural tap was clinically indicated. Lung aspirates were not obtained. CXRs were obtained from cases at the earliest opportunity after admission and interpreted by a trained reading panel.^22^ CXRs were classified as abnormal if there was presence of any of consolidation, pleural effusion or other infiltrate. Abnormal CXRs are referred to here as CXR positive. All samples collected in cases were also collected in controls, with the exception of blood for culture, pleural fluid and IS. Data on antibiotic use were available from clinical records and serum bioassay results.

Study staff were trained on clinical examination and sample collection using a standardized training curriculum.^23^ Samples were processed and analyzed using standardized laboratory methods.^24^ Data-derived density thresholds were applied to PCR results for *S. pneumoniae, H. influenzae, Pneumocystis jirovecii* and cytomegalovirus to optimize sensitivity and specificity.^25–27^

At discharge, a nonstudy clinician recorded up to 2 discharge diagnoses (primary and secondary) into a standardized computer record, selecting the most appropriate diagnoses from a predefined list. Cases of pneumonia were classified here as lower respiratory tract infections (LRTI). Cases of bronchiolitis were classified separately. A senior clinician audited all the discharge diagnoses to ensure consistency of recordings.

### Statistical Analysis and Data Management

Electronic clinical data were exported monthly to a central coordinating center which undertook data management and statistical analyses for all participating sites.^28^

The fraction of pneumonia due to each pathogen was estimated using the PERCH Integrated Analysis (PIA) method, which is described in detail elsewhere.^29–31^ In brief, the PIA is a Bayesian nested partially latent class analysis that integrates the results for each case from blood culture, NP/OP PCR, whole blood PCR for pneumococcus and IS culture for MTB. The PIA also integrates test results from controls to account for imperfect test specificity of NP/OP PCR and whole blood PCR. All analyses were adjusted for age (<1 versus ≥1 year) to account for differences in pathogen prevalences by this factor. The model assumes that each child’s pneumonia was caused by a single pathogen. The PIA estimated both the individual- and population-level etiology probability distributions, each summing to 100% across pathogens where each pathogen has a probability ranging from 0% to 100%. The population-level etiologic fraction estimate for each pathogen was approximately the average of the individual case probabilities and was provided with a 95% credible interval (95% CrI), the Bayesian analogue of the confidence interval. The sensitivity priors used in the model are presented in Table, Supplemental Digital Content 11 (http://links.lww.com/INF/E465). Odds ratios (OR) and 95% confidence intervals (CI) of pathogens detected on NP/OP PCR in cases compared with controls were calculated using logistic regression adjusted for age in months and presence of all other pathogens detected on NP/OP PCR to account for associations between pathogens. Because the WHO clinical case definitions have relatively poor specificity for true lung infection, the primary etiology analysis was restricted to pneumonia cases with abnormal CXRs. We also restricted to HIV-uninfected cases because the etiology of pneumonia is different in HIV-positive cases.^32^

A separate integrated etiology model was run with in-hospital vital status as a covariate, adjusting for the child’s age. This links the mortality status of the child with the etiology assigned at each iteration of the analysis. To calculate the case fatality ratio (CFR) of respiratory syncytial virus (RSV), the proportion of children who died among all those assigned to RSV is calculated at each iteration of the analysis, and those CFRs are averaged across all iterations to obtain an overall CFR and the distribution of the iteration-specific CFRs is used to calculate a 95% CrI. This is similarly done for all the remaining (non-RSV) cases at each iteration to obtain a non-RSV CFR.

We described the primary hospital-assigned discharge diagnoses among all admissions (28 days to 59 months of age) and among PERCH cases and used LRTI recorded in either primary or secondary discharge diagnoses to estimate specificity of the PERCH pneumonia case definition.

Descriptive statistical analyses were performed using SAS 9.3 (SAS Institute, Cary, NC), and the integrated etiology analysis was performed using R Statistical Software 3.3.1 (The R Development Core Team, Vienna, Austria) and Bayesian inference software JAGS 4.2.0 (http://mcmc-jags.sourceforge.net/). The R package used to perform the PERCH Integrated Analysis, named the Bayesian Analysis Kit for Etiology Research, is publicly available at https://github.com/zhenkewu/baker.

### Ethics

We obtained written informed consent from parents or legal guardians of all participants. The Kenya Medical Research Institute Ethical review committee and the Oxford University Tropical Research Ethics Committee approved the study.

## RESULTS

### Study Participants

**FIGURE 1.**
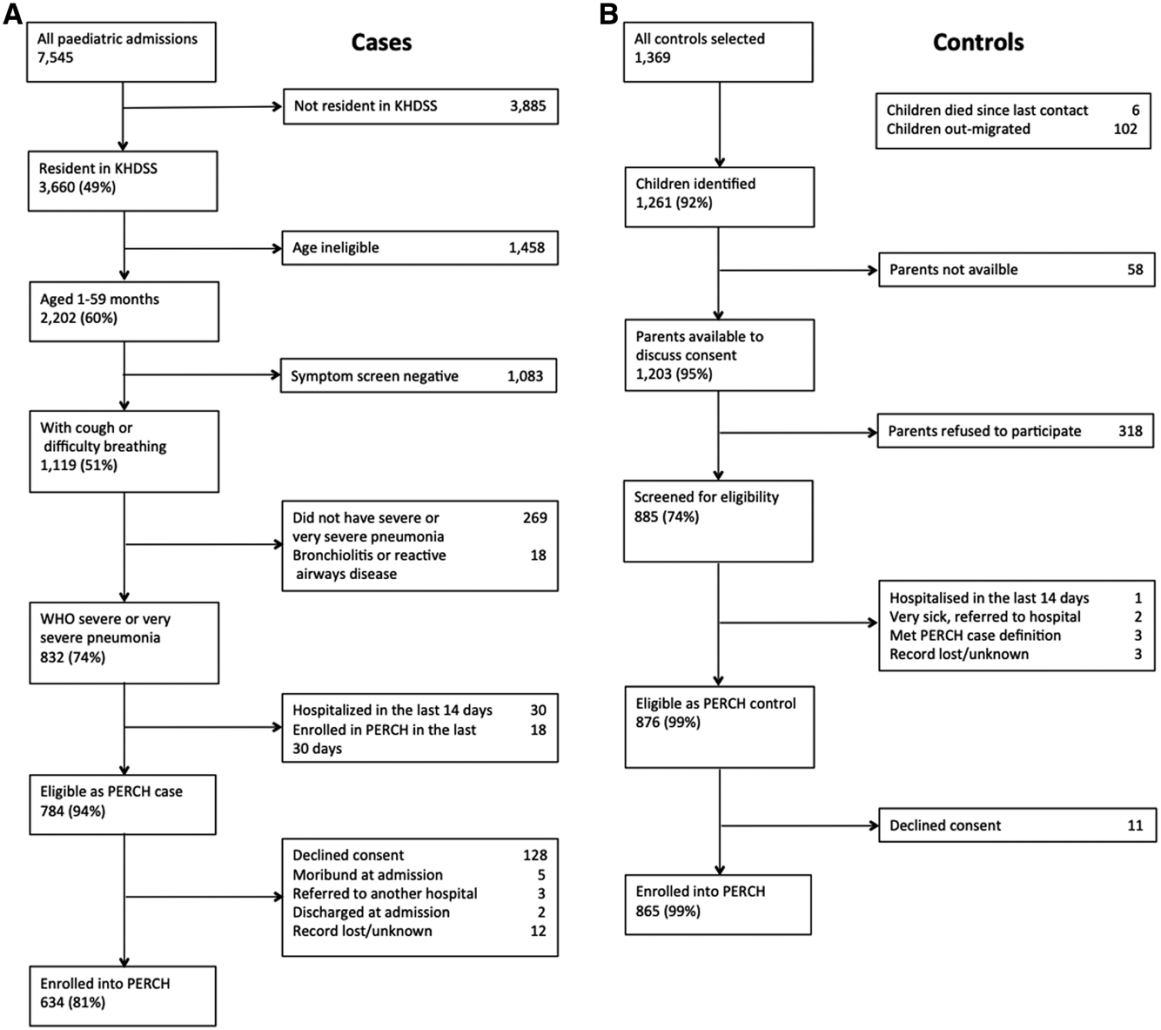
Recruitment flow chart. Left, Case recruitment. Right, Control recruitment.

Of 7545 children admitted to KCH in the study period, 2202 were KHDSS residents 28 days to 59 months of age; of these, 832 had severe or very severe pneumonia, 784 were eligible for inclusion in PERCH, and 634 were enrolled (Fig. 1). The proportion who agreed to participate varied by location (72%–100%) and was lowest among urban dwellers in Kilifi town (Table, Supplemental Digital Content 2, http://links.lww.com/INF/D872). Among those who were eligible, those who were enrolled did not differ by age, sex and pneumonia defining features or in-hospital mortality from those who were not (Table, Supplemental Digital Content 3, http://links.lww.com/INF/D873). The incidence of enrolment to PERCH was higher in locations located closer to KCH (Figure, Supplemental Digital Content 4, http://links.lww.com/INF/D874).

Of 1369 children invited to participate as controls, 889 (65%) were screened and 877 (64%) were eligible, of whom 865 (99%) were enrolled (Fig. 1). Controls were well matched to cases on age and season (Table 1).

**TABLE 1.** Demographic Characteristics of HIV-Uninfected Cases and Controls

	All Cases (N = 630*)	CXR Abnormal† (N = 282*)	CXR Normal (N = 284*)	Controls (N = 863*)
	n (%)	n (%)	n (%)	n (%)
Age				
28 days to 5 m	209 (33)	90 (32)	86 (30)	234 (27)
6–11 m	130 (21)	66 (23)	51 (18)	190 (22)
12–23 m	167 (27)	79 (28)	78 (28)	247 (29)
24–59 m	124 (20)	47 (17)	69 (24)	192 (22)
Sex				
Male	369 (59)	152 (54)	179 (63)	452 (52)
Female	261 (41)	130 (46)	105 (37)	410 (48)
Season of enrolment				
Rainy (Apr to Jul, Oct to Dec)	374 (60)	166 (59)	167 (59)	478 (55)
Dry (Jan to Mar, Aug to Sep)	256 (41)	116 (41)	117 (41)	385 (45)
HIV exposure status				
Exposed‡	71 (11)	34 (12)	30 (11)	144 (17)
Unexposed	559 (89)	248 (88)	254 (89)	719 (83)
Malaria slide				
Negative	583 (93)	271 (97)	252 (89)	797 (98)
Positive	43 (7)	9 (3)	30 (11)	15 (2)
Mid upper arm circumference§				
≥11.5 cm	370 (89)	166 (87)	178 (90)	611 (99)
<11.5 cm	48 (12)	24 (13)	19 (10)	8 (1)
Weight-for-height Z scores				
>−2	425 (70)	177 (66)	207 (75)	801 (94)
−3 to −2	105 (18)	56 (21)	36 (13)	30 (4)
<−3	74 (12)	36 (13)	33 (12)	22 (3)
Weight-for-age Z scores				
>−2	358 (57)	146 (52)	172 (61)	710 (83)
−3 to −2	131 (21)	65 (23)	56 (20)	109 (13)
<−3	135 (22)	68 (24)	53 (19)	38 (4)
Height-for-age Z scores				
>−2	383 (63)	158 (58)	184 (66)	508 (59)
−3 to −2	124 (20)	68 (25)	48 (17)	173 (20)
<−3	104 (17)	47 (17)	47 (17)	178 (21)
PCV10 vaccination status¶				
None	53 (9)	14 (5)	24 (9)	34 (4)
Fully vaccinated (<1 year)	287 (85)	136 (87)	117 (85)	392 (93)
Fully vaccinated (≥1 year)	211 (75)	93 (75)	106 (75)	330 (78)
Pentavalent vaccination status‖				
None	40 (6)	11 (4)	16 (6)	13 (2)
Fully vaccinated (<1 year)	277 (82)	133 (86)	111 (81)	387 (92)
Fully vaccinated (≥1 year)	273 (97)	117 (95)	141 (99)	405 (96)
Serum antibiotic activity				
No	485 (87)	233 (90)	207 (86)	754 (97)
Yes	73 (13)	26 (10)	35 (15)	21 (3)
Parental report of antibiotic use				
No	310 (56)	125 (52)	157 (62)	839 (97)
Yes	242 (44)	116 (48)	98 (38)	24 (3)
Prior exposure to antibiotics**				
No	317 (58)	155 (61)	132 (56)	752 (97)
Yes	229 (42)	99 (39)	104 (44)	21 (3)

*Restricted to HIV-uninfected participants.

†CXR abnormal defined as chest radiograph finding: consolidation or any other infiltrate.

‡Maternal report of being HIV positive during pregnancy and ELISA results either missing, negative or positive without confirmatory virology test results.

§Restricted to children ≥6 months of age.

¶For children <1 year of age, defined as ≥1 dose and up-to-date for age at enrollment, doses received, and Kenya EPI schedule (allowing 4-week window each for dose). For children ≥1 year of age, defined as 2 doses if given ≥8 weeks apart and child was >1 year of age at first dose; 1 dose if age at any dose or age at introduction was >2 years.

‖For children <1 year of age, defined as ≥1 dose and up-to-date for age at enrollment, doses received, and Kenya EPI schedule (allowing 4-week window each for dose). For children ≥1 year of age, defined as 3 doses.

**Defined as serum bioassay positive (cases and controls), antibiotics administered at the referral facility, or antibiotic administration before whole blood specimen collection at the study facility (cases only); restricted to children with blood specimen collected.

### Clinical Characteristics

Four (0.6%) cases and 2 (0.2%) controls were HIV positive and were excluded from further analyses. Clinical characteristics of HIV-uninfected cases are presented in Table 2. CXRs were obtained in 96% (602/630); of the 28 cases without a CXR, 5 died before a CXR was obtained. Eight cases had CXRs obtained >72 hours after admission and were excluded from analyses restricted or stratified by CXR findings. Among the 594 remaining cases, 282 (47%) were CXR positive.

**TABLE 2.** Clinical Characteristics of HIV-Uninfected Cases

	All Cases (N = 630)	CXR Abnormal* (N = 282)	CXR consolidation (N = 119)	CXR Other Infiltrate Only (N = 163)	CXR Normal (N = 284)	Severe Cases (N = 307)	Very Severe Cases (N = 323)	Died in Hospital (N = 33)
	n (%)	n (%)	n (%)	n (%)	n (%)	n (%)	n (%)	n (%)
Age								
28 days to 5 months	209 (33)	90 (32)	49 (41)	41 (25)	86 (30)	114 (37)	95 (29)	15 (46)
6–11 months	130 (21)	66 (23)	20 (17)	46 (28)	51 (18)	73 (24)	57 (18)	5 (15)
12–23 months	167 (27)	79 (28)	26 (22)	53 (33)	78 (28)	68 (22)	99 (31)	9 (27)
24–59 months	124 (20)	47 (17)	14 (12)	23 (14)	69 (24)	52 (17)	72 (22)	4 (12)
Female	261 (41)	130 (46)	58 (49)	72 (44)	105 (37)	126 (41)	135 (42)	20 (61)
Very severe pneumonia	323 (51)	114 (40)	55 (46)	59 (36)	173 (61)	0 (0)	323 (100)	22 (67)
Case fatality								
Died in hospital	33 (5)	17 (6)	12 (10)	5 (3)	9 (3)	11 (4)	22 (7)	33 (100)
Died within 30 days of discharge	6 (1)	1 (0.4)	1 (0.8)	0 (0)	3 (1)	3 (1)	3 (1)	0 (0)
Chest radiograph findings†								
Taken within 72 h	594 (94)	282 (100)	119 (100)	163 (100)	284 (100)	291 (95)	303 (94)	28 (85)
Consolidation	119 (19)	119 (42)	119 (100)	0 (0)	0 (0)	64 (21)	55 (17)	12 (36)
Other infiltrate only	163 (26)	163 (58)	0 (0)	163 (100)	0 (0)	104 (34)	59 (18)	5 (15)
Normal	284 (45)	0 (0)	0 (0)	0 (0)	284 (100)	111 (36)	173 (54)	9 (27)
Uninterpretable	28 (4)	0 (0)	0 (0)	0 (0)	0 (0)	12 (4)	16 (5)	2 (6)
Not taken	28 (4)	0 (0)	0 (0)	0 (0)	0 (0)	10 (3)	18 (6)	5 (15)
Child died	5 (0.8)	0 (0)	0 (0)	0 (0)	0 (0)	1 (0.3)	4 (1)	5 (15)
Other reasons	23 (4)	0 (0)	0 (0)	0 (0)	0 (0)	9 (3)	14 (4)	0 (0)
Clinical characteristics								
Lower chest wall indrawing	492 (78)	259 (92)	110 (92)	149 (91)	186 (65)	307 (100)	186 (57)	28 (85)
Hypoxemia‡	185 (29)	106 (38)	57 (48)	49 (30)	52 (18)	75 (24)	109 (34)	18 (55)
Oxygen use at admission	132 (21)	75 (27)	42 (35)	33 (20)	36 (13)	44 (14)	88 (27)	14 (42)
Tachypnoea	439 (70)	224 (79)	99 (83)	125 (77)	174 (61)	231 (75)	208 (64)	25 (76)
Tachycardia	349 (55)	166 (59)	77 (65)	89 (55)	145 (51)	165 (54)	184 (57)	18 (55)
Central cyanosis	5 (0.8)	1 (0.4)	0 (0)	1 (0.6)	3 (1)	0 (0)	5 (2)	3 (9)
Convulsions	89 (14)	16 (6)	4 (3)	12 (7)	61 (22)	1§ (0.3)	88 (27)	5 (15)
Lethargy	144 (23)	55 (20)	27 (23)	28 (17)	72 (25)	0 (0)	144 (45)	13 (39)
Unable to feed	78 (12)	36 (13)	22 (19)	14 (9)	28 (10)	0 (0)	67 (21)	3 (9)
Vomiting	67 (11)	14 (5)	6 (5)	8 (5)	44 (16)	0 (0)	67 (21)	3 (9)
Crackles	247 (39)	134 (48)	60 (50)	74 (45)	87 (31)	124 (40)	123 (38)	12 (36)
Audible wheeze	29 (5)	13 (5)	4 (3)	9 (6)	15 (5)	19 (6)	10 (3)	1 (3)
Wheeze on auscultation	83 (13)	41 (15)	15 (13)	26 (16)	39 (14)	49 (16)	34 (11)	4 (12)
Grunting	40 (6)	23 (8)	11 (9)	12 (7)	12 (4)	12 (4)	28 (9)	4 (12)
Nasal flaring	317 (50)	166 (59)	76 (64)	90 (55)	119 (42)	162 (53)	155 (48)	20 (61)
Temp >38°C	267 (42)	128 (45)	59 (50)	69 (42)	114 (40)	130 (42)	143 (44)	11 (33)
Leukocytosis	282 (45)	145 (51)	64 (54)	81 (50)	112 (39)	139 (45)	143 (44)	11 (33)
CRP ≥ 40 mg/dL	154 (24)	78 (28)	46 (39)	32 (20)	66 (23)	71 (23)	83 (26)	10 (30)
Hemoglobin								
≤7.5 g/dL	83 (13)	47 (17)	21 (18)	26 (16)	30 (11)	38 (12)	45 (14)	6 (18)
>7.5 g/dL	536 (85)	231 (82)	98 (83)	133 (82)	248 (88)	264 (86)	272 (84)	27 (82)
Malaria slide positive	43 (7)	9 (3)	3 (3)	6 (4)	30 (11)	6 (2)	37 (12)	1 (3)
HIV exposure status								
Exposed	4 (0.6)	1 (0.4)	0 (0)	1 (0.6)	3 (1)	0 (0)	4 (1)	2 (6)
Unexposed	559 (89)	248 (88)	101 (85)	147 (90)	254 (89)	273 (89)	286 (89)	23 (70)
Unknown	67 (11)	33 (12)	18 (15)	15 (9)	27 (10)	34 (11)	33 (10)	8 (24)
MUAC < 11.5 cm‖	48 (12)	24 (13)	8 (11)	16 (13)	19 (10)	21 (11)	27 (12)	6 (33)
Weight-for-height Z scores								
>−2	425 (70)	177 (66)	70 (61)	107 (69)	207 (75)	213 (72)	212 (68)	15 (52)
−3 to ≤−2	105 (18)	56 (21)	28 (25)	28 (18)	36 (13)	48 (16)	57 (18)	7 (24)
<−3	74 (12)	36 (13)	16 (14)	20 (13)	33 (12)	33 (11)	41 (13)	7 (24)
Weight–for-age Z scores								
>−2	358 (57)	146 (52)	54 (45)	92 (58)	172 (61)	178 (59)	180 (56)	7 (22)
−3 to ≤−2	131 (21)	65 (23)	27 (23)	38 (24)	56 (20)	63 (21)	68 (21)	8 (25)
<−3	135 (22)	68 (24)	38 (32)	30 (19)	53 (19)	62 (20)	73 (23)	17 (53)
Height-for-age Z scores								
>−2	383 (63)	158 (58)	61 (53)	97 (62)	184 (66)	185 (63)	198 (63)	9 (29)
−3 to ≤−2	124 (20)	68 (25)	30 (26)	38 (24)	48 (17)	64 (22)	60 (19)	8 (26)
<−3	104 (17)	47 (17)	25 (22)	22 (14)	47 (17)	47 (16)	57 (18)	14 (45)
Duration of illness**								
0–2 days	241 (38)	99 (35)	35 (29)	64 (39)	118 (42)	114 (37)	127 (39)	7 (21)
3–5 days	268 (43)	130 (46)	56 (47)	74 (45)	111 (39)	134 (44)	134 (42)	11 (33)
>5 days	121 (19)	53 (19)	28 (24)	25 (15)	55 (19)	59 (19)	62 (19)	15 (46)
Duration of hospitalization								
0–2 days	196 (31)	68 (24)	23 (19)	45 (28)	106 (37)	93 (30)	103 (32)	15 (45)
3–5 days	303 (48)	138 (49)	53 (45)	85 (52)	141 (50)	151 (49)	152 (47)	8 (24)
>5 days	131 (21)	76 (27)	43 (36)	33 (20)	37 (13)	63 (21)	68 (21)	10 (30)
Antibiotic use								
Prior exposure to antibiotics††	229 (42)	99 (39)	42 (39)	57 (39)	104 (44)	111 (41)	118 (43)	7 (27)
Parental report of antibiotic use before hospitalization	242 (44)	116 (48)	52 (50)	64 (47)	98 (38)	117 (44)	125 (44)	10 (34)

*CXR abnormal defined as consolidation and/or other infiltrate.

†Eight cases had a CXR obtained more than 3 days after enrollment and their CXR findings were not used in analyses. Other reasons no CXR available included child discharged or absconded before CXR (N = 9), operator error (N = 1), transferred to another facility (N = 1) and other reasons (N = 12).

‡Hypoxemia defined as oxygen saturation <92% or on supplemental oxygen if a room air oxygen saturation reading was not available (N = 53).

§Single brief convulsion without other very severe case-defining signs.

¶Elevated white blood cells defined as >15 × 10^9^ cells/L for children 1–11 months and >13 × 10^9^ cells/L for children 12–59 months.

‖Restricted to children ≥6 months of age.

**Duration of illness defined as maximum days of reported symptoms for any of the following: cough, wheeze, fever or difficulty breathing.

††Defined as serum bioassay positive (cases and controls), antibiotics administered at the referral facility, or antibiotic administration before whole blood specimen collection at the study facility (cases only); restricted to children with blood specimen collected.

CRP indicates C-reactive protein; MUAC, mid upper arm circumference.

### Laboratory Results

Blood was cultured in 629 (99.7%) cases; a pathogen was isolated in 10 (1.6%) cases overall and in 9 (3.2%) CXR-positive cases (Table, Supplemental Digital Content 5, http://links.lww.com/INF/D875). These included 5 *S. pneumoniae*, 2 *H. influenzae* nontype b, and one each of *Staphylococcus aureus, Pseudomonas. aeruginosa* (isolated in a child with a normal CXR) and a mixed infection with Salmonella species and *Streptococcus* Group A in 1 child. A contaminant was cultured in 51 (8.1%) samples. In all 4 HIV-positive cases, blood cultures were negative. Three pleural fluid samples were collected: 1 was positive for *S. pneumoniae* by culture and PCR; and 2 were positive for *S. aureus* by culture. Of the 596 IS specimens, 2 (0.3%) were positive for *Mycobacterium tuberculosis* by culture; one of these cases was CXR positive (Table, Supplemental Digital Content 5, http://links.lww.com/INF/D875).

In the NPOP samples, at least 1 pathogen was detected by PCR in 99% (588/628) of cases and 98% (813/855) of controls. The median number of NPOP pathogens detected per case and per control was 4 (IQR: 3–4) and 3 (IQR: 2–4), respectively (Table, Supplemental Digital Content 6, http://links.lww.com/INF/D876). Among 581 cases with available data on time and date of antibiotic administration at admission, NPOP specimens were collected before antibiotic administration in 355 (58%). There was seasonal variation in the isolation of RSV, rhinovirus, human metapneumovirus and parainfluenza virus 1–4 (Figure, Supplemental Digital Content 7, http://links.lww.com/INF/D877). NPOP and whole blood PCR findings are presented in Table, Supplemental Digital Content 8, http://links.lww.com/INF/D878.

### Etiology Results

**FIGURE 2.**
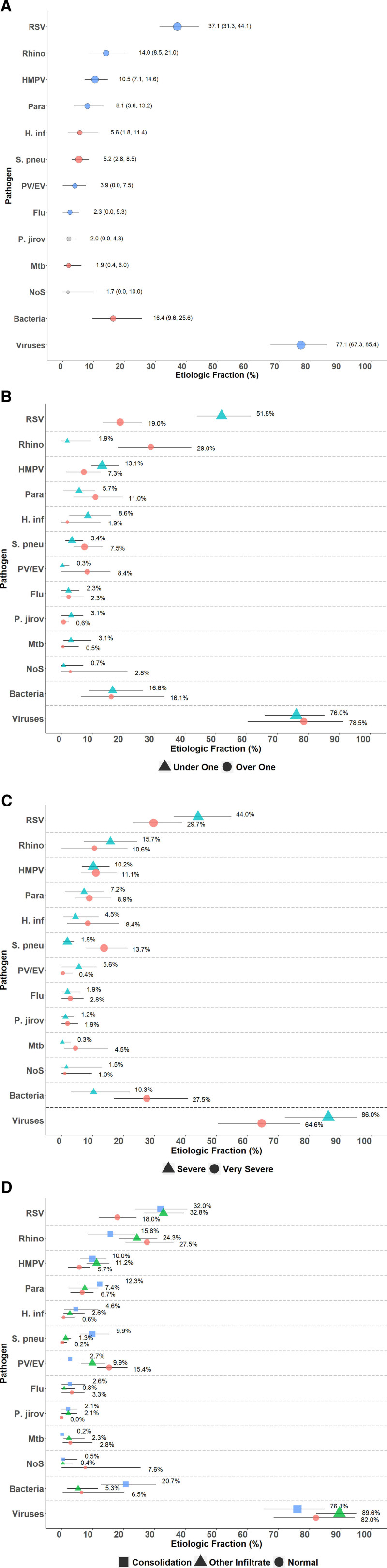
Top 10 causes of radiologically confirmed pneumonia in HIV-uninfected cases, stratified by age, severity and CXR findings. Radiologically confirmed pneumonia defined by the presence of any of consolidation, pleural effusion or other infiltrate on chest radiograph. Description of symbols: Line represents the 95% credible interval. The size of the symbol is scaled based on the ratio of the estimated etiologic fraction to its standard error. Of 2 identical etiologic fraction estimates, the estimate associated with a larger symbol is more informed by the data than the priors. Panel C Stratified by WHO 2005 pneumonia case classification: severe pneumonia, cough or difficulty in breathing and lower chest wall indrawing without any danger sign; and very severe pneumonia, cough or difficulty in breathing and any danger sign. Flu indicates influenza virus A, B and C; H. inf, *Haemophilus influenzae*; HMPV, human metapneumovirus A/B; Mtb, *Mycobacterium tuberculosis*; NoS, not otherwise specified (ie, pathogens not tested for); P. jirov, *P. jirovecii*; Para, parainfluenza virus types 1, 2, 3 and 4; PV/EV, parechovirus/enterovirus; Rhino, Human rhinovirus; S. pneu, *Streptococcus pneumoniae*.

In the PIA model, the main causes of CXR-positive pneumonia were viruses (77%; 95% CrI: 67%–85%), with RSV being the most common (37%; 95% CrI: 31%–44%, Fig. 2A–2D and Table, Supplemental Digital Content 9, http://links.lww.com/INF/D879). *H. influenzae* (6%; 95% CrI: 2%–11%) and *S. pneumoniae* (5%; 95% CrI: 3%–9%) were the most common bacterial causes (Fig. 2A and Table, Supplemental Digital Content 9, http://links.lww.com/INF/D879). Among those over 1 year of age, rhinovirus was the most common pathogen (29%; 95% CrI: 18%–42%, Fig. 2B and Table, Supplemental Digital Content 9, http://links.lww.com/INF/D879). The CFRs for RSV and non-RSV cases were 1.6 (95% CrI: 0–4.4) and 8.1 (95% CrI: 6.4–9.6), respectively.

### Discharge Diagnoses

**FIGURE 3.**
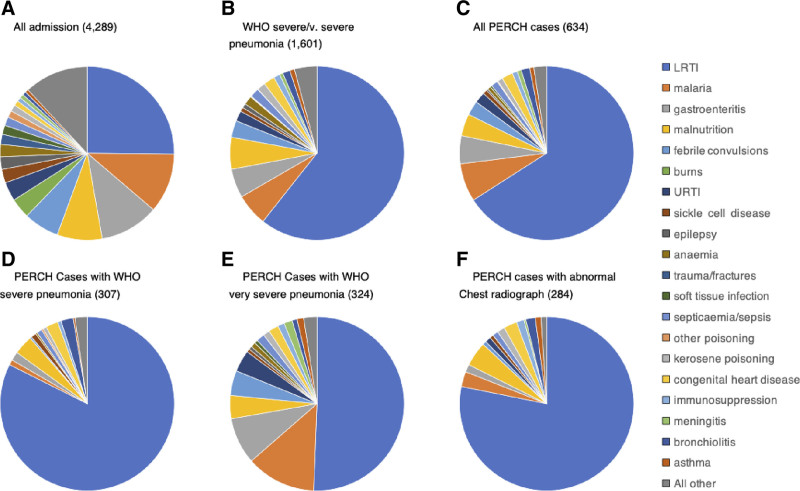
Primary discharge diagnoses for children 28 days to 59 months of age admitted at the study hospital during the PERCH study. WHO Pneumonia classification (2005): severe pneumonia, cough or difficulty in breathing and lower chest wall indrawing without any danger sign; and very severe pneumonia, cough or difficulty in breathing and any danger sign. Abnormal chest radiograph: presence of any of consolidation, pleural effusion or other infiltrate on chest radiograph. Discharge diagnoses (primary and secondary) are recorded in a standard computer form at discharge by nonstudy clinicians during discharge. The most appropriate diagnoses are selected from a predefined list based on the diagnosis recorded during the course of the admission. At the study hospital bronchiolitis is differentiated at discharge diagnosis as part of a longitudinal surveillance program in Kilifi for RSV. In PERCH, children with wheeze/bronchiolitis were excluded from the study only if their case-defining symptoms (usually lower chest wall indrawing) resolved after bronchodilator treatment.

LRTI was a primary discharge diagnosis in 25% of all admissions and 66% of WHO-defined severe or very severe PERCH cases (Fig. 3). Among PERCH cases, 28% (177/634) were not assigned LRTI in either their primary or secondary discharge diagnosis (Table, Supplemental Digital Content 10, http://links.lww.com/INF/D880). The percentage without LRTI was higher for very severe pneumonia (42%, 135/324) than for severe pneumonia (14%, 42/310, *P* < 0.005). Among 284 CXR-positive cases, 42 (15%) did not have a discharge diagnosis of LRTI (Fig. 3 and Table, Supplemental Digital Content 10, http://links.lww.com/INF/D880), including 23 (8%) whose CXRs showed consolidation or effusion according to the radiologic reading standard.^33^

## DISCUSSION

Using an integrated analysis of all available etiology data, we found that viruses accounted for 77% of the attribution of CXR-positive pneumonia among HIV-uninfected children with WHO-defined severe or very severe pneumonia in Kilifi, Kenya. RSV and rhinovirus were the commonest viral causes accounting for 37% and 14%, respectively. *S. pneumoniae* and *H. influenzae*, which were the main causes of radiologically confirmed pneumonia (consolidation with or without pleural effusion) in the prevaccine era, accounted for only 11% of CXR-positive cases (consolidation, infiltrates or pleural effusion) in our study.

In the Kilifi PERCH site, and also in Bangladesh, the proportion of pneumonia attributed to viruses, 77% and 78%, respectively, was substantially higher than the average across the 7 PERCH sites (61%).^31^ We undertook a pilot study for the PERCH methods in Kilifi among children 1–59 months of age between January and December 2010.^34^ In the pilot study, a cause was determined in only 33% of pneumonia cases: virus-only infections in 25%, bacterial only in 6% and mixed viral-bacterial infections in 1% of cases in an analysis of blood culture and NP sample results. The pilot study was typical of previous etiology research in that children with no positive tests were designated “unknown cause” and in our study they accounted for 67% of all cases investigated. In the main study, we assigned each child an equal prior probability of etiology for each of the 33 pathogens tested by PCR and generated posterior probabilities based on the individual pattern of test results so that a cause was assigned for every case. This novel approach provides an indication of viral etiology in a much larger proportion of cases. This, in turn, presents a clinical challenge to identify, at presentation, the minority of children with a bacterial cause in order to target antibiotic treatment appropriately.

Compared with the pilot study, the main study also illustrates a significant change in the blood culture test results; the yield of pneumococci from blood cultures fell from 4% in the pilot study to 0.8% in the main study. Although blood culture is an insensitive method to define etiology, the reduction in test positivity is consistent with the declining incidence of *S. pneumoniae* disease following pneumococcal vaccine introduction.^6,7^ PCV10 was introduced in January 2011, immediately after the pilot study terminated, with a catch-up campaign that reached two-thirds of all children <5 years of age in Kilifi County by March 2011^7^; the PERCH study began in August 2011. Interrupted time series analyses, centered on PCV10 introduction, estimated that the incidence of admission with WHO-defined severe or very severe pneumonia and radiologically confirmed pneumonia declined by 27% and 48%, respectively.^7^ Of note, the radiologic definition in this impact study was restricted to consolidation and/or effusion,^7,35^ whereas the definition used in PERCH also included abnormal infiltrates. However, given these substantial changes in pneumonia incidence attributable to a single pathogen vaccine, it is not surprising that the contribution of bacteremic pneumococcal pneumonia appears considerably reduced in PERCH compared with the pilot study.

RSV was the commonest viral cause of pneumonia cases in Kilifi and in all of the other PERCH sites. Previous studies in Kilifi identified an association between RSV infection in the nasopharynx and admission with pneumonia^34,36^; the prevalence of nasopharyngeal infection was 12%–27% among hospitalized pneumonia cases, 16% among outpatient controls with upper respiratory tract infections and 2% among healthy controls.^16,34,36^ RSV infections are associated with low (2.4%) mortality at KCH.^16^ In the PERCH study in Kilifi, the CFRs (estimated using the integrated analysis) for RSV and for all other pneumonias were 1.6% (95% CrI: 0–4.4) and 8.1% (95% CrI: 6.4–9.6), respectively. Although 37% of pneumonia was attributed to RSV, the low CFR estimate suggests that RSV is responsible for only a small fraction of pneumonia deaths in Kilifi.

Rhinovirus was a common infection at all sites with a prevalence of 17%–36% in cases and 14%–26% in controls. However, it was significantly associated with case status in only 2 countries; Kenya (OR 2.4, 95% CI 1.6, 3.7) and Bangladesh (OR 2.5, 95% CI 1.7, 3.7). The prevalence of rhinovirus varies across time and place, as does the evidence implying a causal role. In the Kilifi pilot study, for example, the prevalence of rhinovirus in NP specimens was 23% and 22% in hospitalized cases of WHO-defined pneumonia and outpatient controls without pneumonia, respectively (OR 1.0, 95% CI 0.7, 1.3).^34^ In the South Africa site of the PERCH study, the prevalence of rhinovirus in cases and controls was 19% and 22%, respectively (OR = 1.0, 95% CI: 0.5–1.9). However, in a separate study in South Africa at the same time, rhinovirus was found in 40% of children <5 years of age admitted with severe acute respiratory illness and 33% of outpatient controls without respiratory symptoms (aRR = 1.8, 95% CI: 1.4–2.3).^37^ It is not yet clear whether rhinovirus is a direct lung pathogen or acts synergistically to precipitate pneumonia caused by other pathogens or is falsely implicated by confounding. For example, children from crowded households are more likely than those from uncrowded households to be exposed to infections of the upper and of the lower respiratory tract, generating an association between crowding and pneumonia and an independent association between crowding and pathogens in the upper respiratory tract.

The WHO clinical definitions of pneumonia were designed to favor sensitivity over specificity in order to make life-saving antibiotics widely accessible. In the primary PERCH analysis, we increased our specificity by analyzing only cases with abnormal CXRs. In Kilifi, we also explored the specificity of these case definitions through an analysis of the discharge diagnoses among different sub-groups of PERCH patients (Table, Supplemental Digital Content 10, http://links.lww.com/INF/D880). These discharge diagnoses were not part of the PERCH protocol and no attempt was made to standardize interpretations or validate the results. Clinicians identified non-LRTI conditions in the discharge diagnosis in over a quarter of all PERCH cases, rising to 42% among very severe pneumonia cases. Common primary discharge diagnoses for these patients were malaria (7%), gastroenteritis (5%), malnutrition (4%), febrile convulsions (3%) and upper respiratory tract infection (2%) illustrating the considerable overlap in clinical presentations between common pediatric conditions and the limitations of a case definition focused on admission features alone. The clinicians also did not mention LRTI in the discharge diagnosis of 42 PERCH cases later classified as having abnormal CXRs by the reading panel, 19 of these had infiltrates only (Table, Supplemental Digital Content 10, http://links.lww.com/INF/D880). Some of the diagnoses given, including paraffin poisoning (n = 3) and bronchiolitis (n = 4), are compatible with CXR abnormalities. Some children with predisposition to recurrent pneumonia due to chronic conditions (eg, congenital heart disease, malnutrition, sickle cell disease) may have had CXR changes from previous pneumonia episodes.^38^ The attending pediatrician may have interpreted the radiographs as negative for pneumonia. Alternatively, the diagnoses selected may have simply “trumped” pneumonia in a system that only allows the clinician to specify 2 conditions: combinations of malnutrition, malaria, anemia, febrile convulsions, pulmonary TB, immunosuppression, sepsis, sickle cell disease or meningitis account for 15 of these cases. While the WHO clinical case definitions are essential in case management, they do not always capture the dominant pathology or illness in a sick child.

Two limitations of this study are noteworthy. The first is its focus on hospitalized cases.^39^ Studies from Kilifi have already documented poor access to care: for example, approximately 40% of very severe pneumonia cases occurring in KHDSS do not seek care at the study hospital^40^ and 64% of all childhood deaths occur outside the hospital.^18^ The etiology of pneumonia among cases that do not seek care may differ markedly from the patterns documented here. Second, available pneumonia etiology evidence and analytic methods may underestimate the role of bacteria in pneumonia etiology. Much of the pathologic evidence for etiology in pneumonia studies comes from nasopharynx,^34,41–43^ which is frequently colonized by organisms in the absence of pneumonia. In Kilifi, we have little evidence from the anatomic site of pathology as we cultured only 3 pleural fluid aspirates and no lung aspirates. Blood cultures were performed in most children and the PIA model does adjust for sensitivity and the impact of antibiotics, which were measured using a bioassay of serum.^29^ However, the biologic detection of antibiotic exposure is insensitive to antibiotics administered ≥8 hours previously because of their short serum half-life. Parental reports of antibiotic administration were much higher than antibiotics detected in the serum (Table 1), but parental report is also not a reliable measure.

The PERCH study integrated results from multiple sample sites from both cases and controls using partial latent class analysis and a Bayesian analytical approach to provide an estimate of pneumonia etiology in children in Kilifi. Based on these analyses, 77% of the etiology of pneumonia was attributable to viruses, particularly RSV and rhinovirus. This emphasizes the need to advance vaccines against RSV in children and to understand the pathogenesis of pneumonia involving viral infections. It also significantly reduces the likelihood that antibiotics will alter the course of pneumonia illness in a hospitalized child and sets out a significant challenge to identify the minority of hospitalized cases who do require antibiotics. The prevalence of bacteremic pneumococcal pneumonia has declined between the pilot study in 2010 and the main PERCH study in 2011 to 2013 and this can probably best be explained by the significant impact of PCV10 on the incidence of pneumococcal pneumonia.

## ACKNOWLEDGMENTS

We would like to thank all the patients and families that participated in this study. We acknowledge the work of all PERCH Contributors who were involved in data collection at the study site, the clinical and laboratory staff at Kilifi County Hospital and the KEMRI Wellcome Trust Research Programme, Kilifi, Kenya, the central laboratories, members of the PERCH Chest Radiograph Reading Panel, and Shalika Jayawardena and Rose Watt from Canterbury Health Laboratories. We also acknowledge the substantial contributions of members of the PERCH Study Group: Johns Hopkins Bloomberg School of Public Health, Baltimore, MD: Orin S. Levine (Former PI, current affiliation Bill & Melinda Gates Foundation, Seattle, WA), Andrea N. DeLuca, Amanda J. Driscoll, Nicholas Fancourt, Wei Fu, E. Wangeci Kagucia, Ruth A. Karron, Mengying Li, Daniel E. Park, Qiyuan Shi, Zhenke Wu, Scott L. Zeger; The Emmes Corporation, Rockville, MD: Nora L. Watson; Nuffield Department of Clinical Medicine, University of Oxford, United Kingdom: Jane Crawley; Medical Research Council, Basse, The Gambia: Stephen R. C. Howie (site PI); KEMRI-Wellcome Trust Research Programme, Kilifi, Kenya: J. Anthony G. Scott (site PI and PERCH co-PI, joint affiliation with London School of Hygiene and Tropical Medicine, London, United Kingdom); Division of Infectious Disease and Tropical Pediatrics, Department of Pediatrics, Center for Vaccine Development, and Global Health University of Maryland School of Medicine, Baltimore, MD and Centre pour le Développement des Vaccins (CVD-Mali), Bamako, Mali: Karen L. Kotloff (site PI); Medical Research Council: Respiratory and Meningeal Pathogens Research Unit and Department of Science and Technology/National Research Foundation: Vaccine Preventable Diseases, University of the Witwatersrand, Johannesburg, South Africa: Shabir A. Madhi (site PI); International Centre for Diarrhoeal Disease Research, Bangladesh (icddr,b): W. Abdullah Brooks (site PI); Thailand Ministry of Public Health—US CDC Collaboration, Nonthaburi, Thailand: Henry C. Baggett (site PI), Susan A. Maloney (former site PI); Department of Global Health, Boston University School of Public Health, Boston, Massachusetts and University Teaching Hospital, Lusaka, Zambia: Donald M. Thea (site PI); Canterbury Health Laboratories, Christchurch, New Zealand: Trevor P. Anderson, Joanne Mitchell. This paper is published with the approval of the Director, Kenya Medical Research Institute.

## Supplementary Material


