# Supplementary material for: The Etiology of Pneumonia in HIV-uninfected Children in Kilifi, Kenya: Findings From the Pneumonia Etiology Research for Child Health (PERCH) Study
Source: Pediatr Infect Dis J. 2021 Aug 25;40(9):S29–39. doi: 10.1097/INF.0000000000002653 (PMC8448399; doi:10.1097/INF.0000000000002653)
Supplement: Supplementary file 1 [file inf-40-s29-s001.docx]

Supplemental Digital Content 1: PERCH Study Inclusion and exclusion criteria

| ***Inclusion criteria:*** | |
| --- | --- |
| - Admitted to Kilifi County Hospital | |
| - Age 28 days – 59 months | |
| - 2005 WHO defined severe or very severe pneumonia:   Cough or Difficulty in breathing plus any one of the following signs or symptoms: | |
| ***Severe pneumonia***  Lower chest wall indrawing | ***Very severe pneumonia***  Central cyanosis  Difficulty in breastfeeding or drinking^a^  Vomiting everything  Convulsions^b^  Lethargy^c^ or unconsciousness  Head nodding |
| ***Exclusion criteria:*** | |
| - Hospitalized for any cause within the last 14 days - Hospitalized as a PERCH study patient in the past 30 days - Resolution of lower chest wall indrawing in response to bronchodilator therapy in a child with wheeze | |

1. Unable to feed or not feeding well (in an infant who was previously feeding well)
2. More than 1 convulsion in the last 24 hours or one or more prolonged convulsions (15 minutes or more) in this illness
3. An infant who does not wake up on stimulation or, on waking, subsequently moves only on stimulation or does not move at all
