# Supplementary material for: The Etiology of Pneumonia in HIV-uninfected Children in Kilifi, Kenya: Findings From the Pneumonia Etiology Research for Child Health (PERCH) Study
Source: Pediatr Infect Dis J. 2021 Aug 25;40(9):S29–39. doi: 10.1097/INF.0000000000002653 (PMC8448399; doi:10.1097/INF.0000000000002653)
Supplement: Supplementary file 2 [file inf-40-s29-s002.docx]

Supplemental Digital Content 2: Proportion of eligible cases and control participants who consented by location of residence

| **Location** | **Cases^a^** | | | **Controls^a^** | | |
| --- | --- | --- | --- | --- | --- | --- |
|  | **Eligible (N=771)** | **Consented (n=629)** | | **Eligible (N=865)** | **Consented (n=854)** | |
|  | **N** | **n** | ***%*** | **N** | **n** | ***%*** |
| Gede | 7 | 7 | *100* | 40 | 40 | *100* |
| Matsangoni | 24 | 21 | *88* | 61 | 61 | *100* |
| Roka | 36 | 30 | *83* | 65 | 64 | *99* |
| Ngerenya | 45 | 38 | *84* | 67 | 64 | *96* |
| Sokoke | 24 | 22 | *92* | 28 | 28 | *100* |
| Jaribuni | 17 | 14 | *82* | 16 | 16 | *100* |
| Tezo | 96 | 74 | *77* | 74 | 73 | *99* |
| Kilifi township | 207 | 148 | *72* | 56 | 54 | *96* |
| Takaungu Mavueni | 86 | 74 | *86* | 81 | 79 | *98* |
| Kauma | 30 | 26 | *87* | 30 | 30 | *100* |
| Ziani | 39 | 35 | *90* | 89 | 88 | *99* |
| Chasimba | 52 | 47 | *90* | 85 | 84 | *99* |
| Banda ra salama | 21 | 19 | *91* | 38 | 38 | *100* |
| Junju | 73 | 60 | *82* | 106 | 106 | *100* |
| Mtwapa | 14 | 14 | *100* | 29 | 29 | *100* |

^a^ Participants with location data available

χ^2^ test for equal proportions of those who agreed to participate: cases = 0.014, controls = 0.638
