# Supplementary material for: The Etiology of Pneumonia in HIV-uninfected Children in Kilifi, Kenya: Findings From the Pneumonia Etiology Research for Child Health (PERCH) Study
Source: Pediatr Infect Dis J. 2021 Aug 25;40(9):S29–39. doi: 10.1097/INF.0000000000002653 (PMC8448399; doi:10.1097/INF.0000000000002653)
Supplement: Supplementary file 3 [file inf-40-s29-s003.docx]

Supplemental Digital Content 3: Comparison of cases eligible for PERCH by enrollment status

| ***Characteristic*** | ***Enrolled***  ***(N = 634)*** | | ***Not-enrolled***  ***(N = 150)*** | | ***P-value^a^*** |
| --- | --- | --- | --- | --- | --- |
|  | **n** | **%** | **n** | **%** |  |
| Age |  |  |  |  | 0.154 |
| 28 days-5 months | 209 | *33* | 38 | *25* |  |
| 6-11 months | 130 | *21* | 27 | *18* |  |
| 12-23 months | 171 | *27* | 48 | *32* |  |
| 24-59 months | 124 | *20* | 37 | *25* |  |
| Sex |  |  |  |  | 0.528 |
| Male | 371 | *59* | 92 | *61* |  |
| Female | 263 | *42* | 58 | *39* |  |
| Clinical characteristics |  |  |  |  |  |
| Lower chest wall indrawing | 494 | *78* | 115 | *77* | 0.741 |
| Head nodding | 94 | *15* | 16 | *11* | 0.183 |
| Central cyanosis | 5 | *0.8* | 2 | *1* | 0.525 |
| Inability to feed | 78 | *12* | 19 | *13* | 0.924 |
| Vomiting everything | 69 | *11* | 9 | *6* | 0.074 |
| Lethargy | 147 | *23* | 37 | *25* | 0.715 |
| Convulsions | 89 | *14* | 23 | *15* | 0.683 |
| SPO2 < 92% | 156 | *25* | 37 | *25* | 0.988 |
| In-hospital mortality |  |  |  |  |  |
| Died | 33 | *5* | 12 | *8* | 0.135 |
| Lab results |  |  |  |  |  |
| Malaria slide positive | 44 | *7* |  |  |  |
| Blood culture positive | 10 | *2* |  |  |  |

a. χ^2^ test for equal proportions
