# Supplementary material for: The Etiology of Pneumonia in HIV-uninfected Children in Kilifi, Kenya: Findings From the Pneumonia Etiology Research for Child Health (PERCH) Study
Source: Pediatr Infect Dis J. 2021 Aug 25;40(9):S29–39. doi: 10.1097/INF.0000000000002653 (PMC8448399; doi:10.1097/INF.0000000000002653)

**Supplemental Digital Content 4: Incidence of enrolment to PERCH by location in the Kilifi Health and Demographic Surveillance System area**

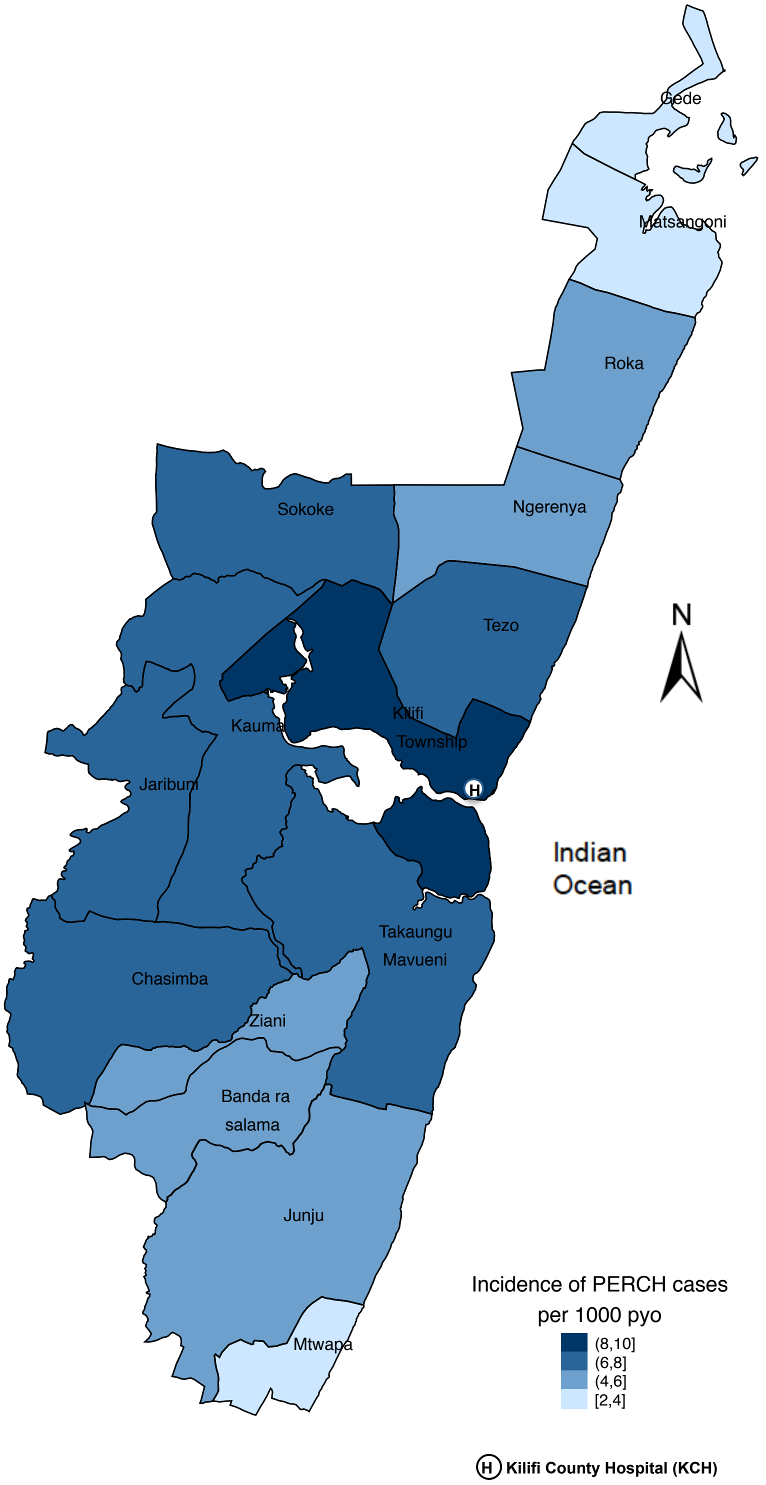

Supplement: Supplementary file 4 [file inf-40-s29-s004.pdf]
