# Supplementary material for: The Etiology of Pneumonia in HIV-uninfected Children in Kilifi, Kenya: Findings From the Pneumonia Etiology Research for Child Health (PERCH) Study
Source: Pediatr Infect Dis J. 2021 Aug 25;40(9):S29–39. doi: 10.1097/INF.0000000000002653 (PMC8448399; doi:10.1097/INF.0000000000002653)
Supplement: Supplementary file 5 [file inf-40-s29-s005.docx]

Supplemental Digital Content 5: Blood culture, Pleural fluid and Induced sputum results for cases

|  | ***All Cases*** | | ***CXR+ Cases*** | |
| --- | --- | --- | --- | --- |
|  | **n** | ***%*** | **n** | ***%*** |
| **A. Blood** | *(N = 629)* | | *((N = 282)* | |
| Total positive^a^ | 10 | *1.6* | 9 | *3.2* |
| *S. pneumoniae* | 5 | *0.8* | 5 | *1.8* |
| *S. pneumoniae*, VT (PCV10) | 3 | *0.5* | 3 | *1.1* |
| *S. pneumoniae*, non-VT (PCV10) | 2 | *0.3* | 2 | *0.7* |
| *H. influenzae* | 2 | *0.3* | 2 | *0.7* |
| *H. influenzae* type b | 0 | *0* | 0 | *0* |
| *H. influenzae* non-type b | 2 | *0.3* | 2 | *0.7* |
| *S. aureus* | 1 | *0.2* | 1 | *0.4* |
| *P. aeruginosa* | 1 | *0.2* | 0 | *0* |
| Salmonella species and *Streptococcus* Group A^b^ | 1 | *0.2* | 1 | *0.4* |
| Contaminants | 51 | *8.1* | 24 | *8.5* |
| **B. Pleural Fluid** | *(N = 3)* | | *(N = 2)* | |
| Total positive^c^ | 3 | *100* | 2 | *100* |
| *S. aureus* | 2 | *50* | 1 | *50* |
| *S. pneumoniae^d^* | 1 | *50* | 1 | *50* |
| **C. Induced Sputum** | *(N = 596)* | | *(N=267)* | |
| *Mycobacterium tuberculosis* | 2 | *0.3* | 1 | *0.4* |

a Excluding contaminants

b Mixed infection

*c S. pneumoniae* and 1 *S. aureus* were obtained within 2 days of admission and 1 *S. aureus* 7 days post admission. Only specimens obtained within 3 days of admission were included in the main analysis.

d *S. pneumoniae* was positive by both culture and PCR (positive for serotype 5 on pleural fluid culture)
