# Supplementary material for: The Etiology of Pneumonia in HIV-uninfected Children in Kilifi, Kenya: Findings From the Pneumonia Etiology Research for Child Health (PERCH) Study
Source: Pediatr Infect Dis J. 2021 Aug 25;40(9):S29–39. doi: 10.1097/INF.0000000000002653 (PMC8448399; doi:10.1097/INF.0000000000002653)
Supplement: Supplementary file 6 [file inf-40-s29-s006.docx]

Supplemental Digital Content 6: Co-detection of NPOP pathogens in cases and controls

|  | ***All Cases***  ***(N=628)*** | | | ***CXR+ Cases***  ***(N=282)*** | | | ***All Controls***  ***(N=855)*** | | |
| --- | --- | --- | --- | --- | --- | --- | --- | --- | --- |
| Number of pathogens detected, any positivity |  | | |  | | |  | | |
| Mean (SD) | 3.6 (1.4) | | | 3.7 (1.4) | | | 3.3 (1.3) | | |
| Median (IQR) | 4 (3,4) | | | 4 (3,5) | | | 3 (2,4) | | |
| Number of pathogens detected, above threshold |  | | |  | | |  | | |
| Mean (SD) | 2.3 (1.2) | | | 2.3 (1.2) | | | 1.9 (1) | | |
| Median (IQR) | 2 (2,3) | | | 2 (2,3) | | | 2 (1,3) | | |
|  |  | | |  | | |  | | |
|  | **n** | ***%*** | | **n** | ***%*** | | **n** | ***%*** | |
| Number of pathogens detected, any positivity |  | |  |  | |  |  | |  |
| 0 | 5 | | *0.8* | 3 | | *1* | 16 | | *2* |
| 1 | 41 | | *7* | 16 | | *6* | 46 | | *5* |
| 2 | 83 | | *13* | 34 | | *12* | 162 | | *19* |
| 3 | 169 | | *27* | 66 | | *23* | 249 | | *29* |
| 4+ | 330 | | *53* | 163 | | *58* | 382 | | *45* |
| Number of pathogens detected, above threshold |  | |  |  | |  |  | |  |
| 0 | 23 | | *4* | 11 | | *4* | 38 | | *4* |
| 1 | 125 | | *20* | 49 | | *17* | 271 | | *32* |
| 2 | 250 | | *40* | 112 | | *40* | 321 | | *32* |
| 3 | 154 | | *25* | 72 | | *26* | 156 | | *18* |
| 4+ | 76 | | *12* | 38 | | *14* | 69 | | *8* |
| Pathogen patterns, any positivity |  | |  |  | |  |  | |  |
| Bacteria only |  | |  |  | |  |  | |  |
| Single bacteria | 18 | | *3* | 9 | | *3* | 31 | | *4* |
| 2 or more bacteria | 59 | | *9* | 18 | | *6* | 163 | | *19* |
| Virus only |  | |  |  | |  |  | |  |
| Single virus | 25 | | *4* | 8 | | *3* | 18 | | *2* |
| 2 or more viruses | 12 | | *2* | 5 | | *2* | 11 | | *1* |
| Bacterial-Viral | 509 | | *81* | 239 | | *85* | 616 | | *72* |
| Pathogen patterns, above threshold |  | |  |  | |  |  | |  |
| Bacteria only |  | |  |  | |  |  | |  |
| Single bacteria | 68 | | *11* | 23 | | *8* | 230 | | *27* |
| 2 or more bacteria | 26 | | *4* | 8 | | *3* | 54 | | *6* |
| Virus only |  | |  |  | |  |  | |  |
| Single virus | 58 | | *9* | 26 | | *9* | 43 | | *5* |
| 2 or more viruses | 30 | | *5* | 15 | | *5* | 21 | | *3* |
| Bacterial-Viral | 422 | | *67* | 198 | | *70* | 469 | | *55* |

Note: Analysis population was restricted to HIV-uninfected cases and controls with available NPOP data.
