# Supplementary material for: The Etiology of Pneumonia in HIV-uninfected Children in Kilifi, Kenya: Findings From the Pneumonia Etiology Research for Child Health (PERCH) Study
Source: Pediatr Infect Dis J. 2021 Aug 25;40(9):S29–39. doi: 10.1097/INF.0000000000002653 (PMC8448399; doi:10.1097/INF.0000000000002653)

Supplemental Digital Content 7: Number of PERCH cases recruited per month that had RSV, rhinovirus (Rhino), human metapneumovirus (HMPV) or parainfluenza virus (Para Flu) detected in NPOP specimens by PCR

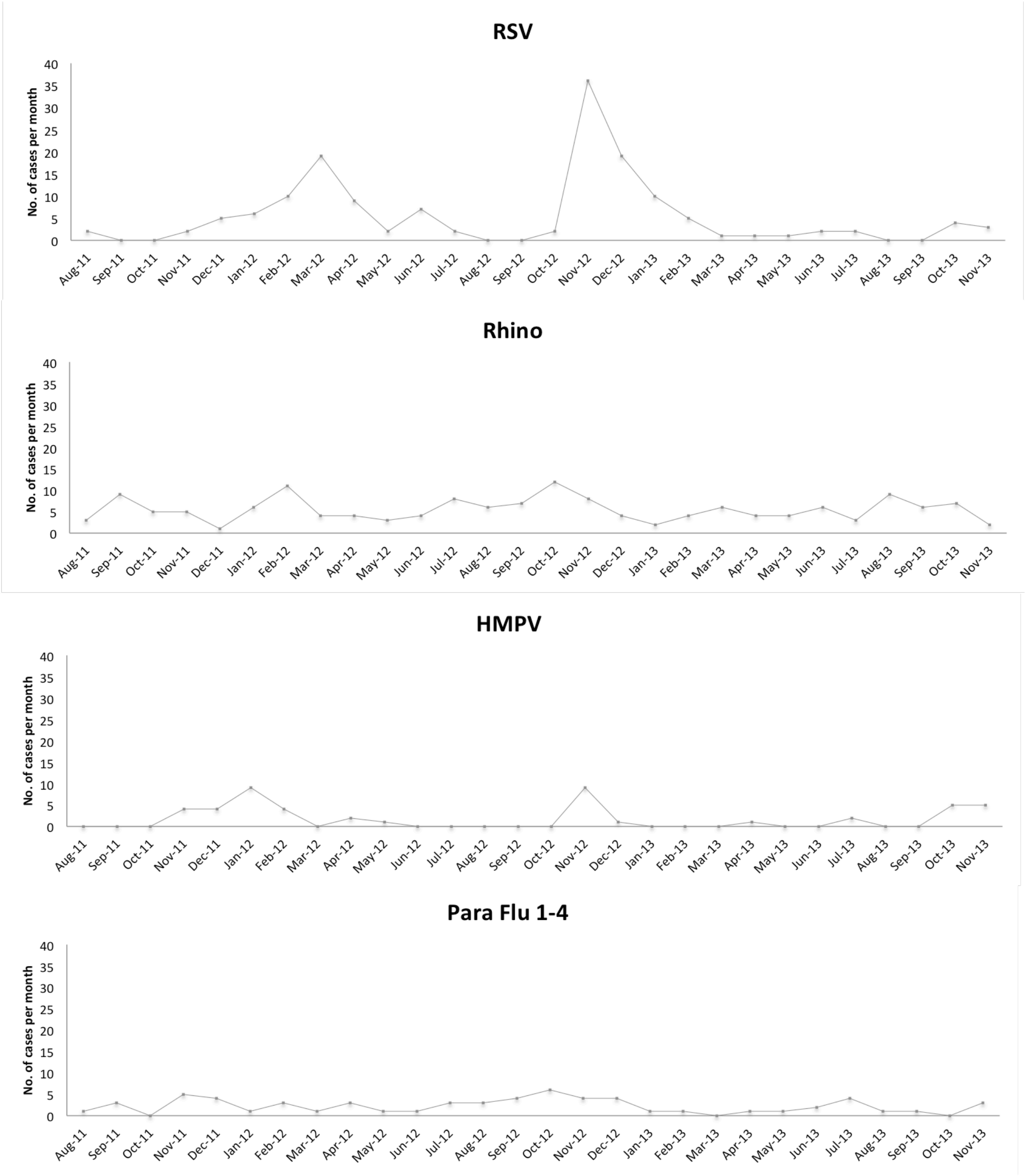

Supplement: Supplementary file 7 [file inf-40-s29-s007.pdf]
