# Supplementary material for: The Etiology of Pneumonia in HIV-uninfected Children in Kilifi, Kenya: Findings From the Pneumonia Etiology Research for Child Health (PERCH) Study
Source: Pediatr Infect Dis J. 2021 Aug 25;40(9):S29–39. doi: 10.1097/INF.0000000000002653 (PMC8448399; doi:10.1097/INF.0000000000002653)
Supplement: Supplementary file 8 [file inf-40-s29-s008.docx]

Supplemental Digital Content 8: NPOP PCR and whole blood *lyt*A PCR for case and control participants

|  | ***All Cases (N = 628)*** | | ***CXR+ Cases***  ***(N=282)*** | | ***All Controls***  ***(N=855)*** | | ***All Cases vs. All Controls^a^*** | | ***CXR+ Cases vs. All Controls^a^*** | | ***All Cases vs. All Controls^b^*** | | ***CXR+ Cases vs. All Controls^b^*** | |
| --- | --- | --- | --- | --- | --- | --- | --- | --- | --- | --- | --- | --- | --- | --- |
|  | ***n*** | ***%*** | ***n*** | ***%*** | ***n*** | ***%*** | ***OR^a^*** | ***95% CI*** | ***OR^a^*** | ***95% CI*** | ***OR^b^*** | ***95% CI*** | ***OR^b^*** | ***95% CI*** |
| **Bacteria** |  |  |  |  |  |  |  |  |  |  |  |  |  |  |
| Any bacteria | 586 | *93* | 266 | *94* | 810 | *95* | 0.80 | 0.52,1.23 | 0.96 | 0.53,1.74 | 1.04 | 0.55,1.98 | 1.28 | 0.53,3.06 |
| Any bacteria, with thresholds applied for *S. pneumoniae* and *H. influenzae* | 516 | *82* | 229 | *81* | 753 | *88* | 0.63 | 0.47,0.84 | 0.60 | 0.41,0.86 | 1.10 | 0.47,2.59 | 1.39 | 0.47,4.16 |
| *S. pneumoniae* |  |  |  |  |  |  |  |  |  |  |  |  |  |  |
| NP/OP, any positivity | 463 | *74* | 214 | *76* | 684 | *80* | 0.72 | 0.56,0.92 | 0.78 | 0.56,1.07 | 0.66 | 0.48,0.91 | 0.70 | 0.44,1.11 |
| NP/OP, ≥ 6.9 log10 copies/ml | 30 | *5* | 14 | *5* | 17 | *2* | 2.52 | 1.38,4.63 | 2.55 | 1.24,5.25 | 2.26 | 1.02,5 | 2.74 | 1.02,7.33 |
| Among those with >6.9 log_10_ copies/ml |  |  |  |  |  |  |  |  |  |  |  |  |  |  |
| PCV10-type | 7 | *23* | 4 | *29* | 2 | *12* | 5.08 | 1.0,24.62 | 6.30 | 1.14,34.77 | 1.29 | 0.05,35.31 | - | - |
| Non PCV10-type | 21 | *70* | 10 | *71* | 14 | *82* | 2.09 | 1.05,4.15 | 2.16 | 0.95,4.94 | - | - | - | - |
| Whole Blood, any positivity | 31 | *5* | 20 | *7* | 48 | *6* | 0.85 | 0.53,1.35 | 1.29 | 0.75,2.22 | - | - | - | - |
| Whole Blood, ≥ 2.2 log10 copies/ml | 18 | *3* | 13 | *5* | 31 | *4* | 0.76 | 0.42,1.37 | 1.28 | 0.66,2.49 | - | - | - | - |
| *H. influenzae* |  |  |  |  |  |  |  |  |  |  |  |  |  |  |
| *H. influenzae* non b | 282 | *45* | 135 | *48* | 314 | *37* | 1.40 | 1.13,1.73 | 1.57 | 1.20,2.06 | 1.21 | 0.92,1.57 | 1.35 | 0.93,1.94 |
| *H. influenzae* non b ≥5.9 log_10_ copies/ml | 96 | *15* | 44 | *16* | 81 | *10* | 1.73 | 1.26,2.37 | 1.79 | 1.20,2.06 | 1.80 | 1.20,2.71 | 1.88 | 1.09,3.23 |
| *H. influenzae* type b | 8 | *1* | 2 | *0.7* | 3 | *0.4* | 3.94 | 1.04,14.95 | 2.10 | 0.35,12.74 | 2.67 | 0.55,13.03 | 3.00 | 0.33,27.44 |
| H*. influenzae* type b ≥5.9 log_10_ copies/ml | 2 | *0.03* | 0 | *0* | 0 | *0* | . | - | - | - | - | - | - | - |
| *S. aureus* | 56 | *9* | 23 | *8* | 60 | *7* | 1.26 | 0.86,1.85 | 1.16 | 0.70,1.92 | 1.59 | 0.98,2.58 | 1.34 | 0.69, 2.63 |
| *C. pneumoniae* | 7 | *1.1* | 5 | *2* | 17 | *2* | 0.59 | 0.24,1.42 | 0.94 | 0.34,2.58 | 0.40 | 0.14,1.18 | 0.61 | 0.16,2.30 |
| *M. catarrhalis* | 481 | *77* | 211 | *75* | 731 | *86* | 0.57 | 0.44,0.74 | 0.51 | 0.37,0.71 | 0.61 | 0.43,0.85 | 0.59 | 0.38,0.92 |
| *M. pneumoniae* | 4 | *0.6* | 2 | *0.7* | 4 | *0.5* | 1.44 | 0.36,5.78 | 1.53 | 0.28,8.43 | 1.25 | 0.25,6.11 | 1.45 | 0.15,13.80 |
| Salmonella species | 4 | *0.6* | 2 | *0.7* | 0 | *0* | - | - | - | - | - | - | - | - |
| Legionella | 0 | *0* | 0 | *0* | 0 | *0* | - | - | - | - | - | - | - | - |
| *B. pertussis* | 1 | *0.2* | 0 | *0* | 0 | *0* | - | - | - | - | - | - | - | - |
| **Fungi** |  |  |  |  |  |  |  |  |  |  |  |  |  |  |
| PCP | 25 | *4.0* | 14 | *5.0* | 38 | *4* | 0.80 | 0.47,1.35 | 1.01 | 0.53,1.91 | 0.98 | 0.51,1.90 | 1.67 | 0.76,3.66 |
| PCP >4 log10 copies/ml | 10 | *2* | 8 | *3* | 9 | *1* | 1.40 | 0.56,3.48 | 2.49 | 0.94,6.55 | 1.82 | 0.69,4.79 | 3.73 | 1.30,10.68 |
| **Viruses^c^** |  |  |  |  |  |  |  |  |  |  |  |  |  |  |
| Any virus | 546 | *87* | 252 | *89* | 645 | *75* | 2.22 | 1.67,2.95 | 2.74 | 1.82,4.14 | 1.10 | 0.73,1.66 | 1.30 | 0.71,2.37 |
| Any virus, above threshold | 510 | *81* | 239 | *85* | 533 | *62* | 2.62 | 2.05,3.35 | 3.30 | 2.31,4.70 | 1.57 | 0.98,2.51 | 2.00 | 1.04,3.86 |
| Adenovirus | 50 | *8* | 19 | *7* | 68 | *8* | 1.08 | 0.73,1.59 | 0.87 | 0.51,1.48 | 1.45 | 0.92,2.29 | 1.44 | 0.77,2.70 |
| CMV | 283 | *45* | 136 | *48* | 427 | *50* | 0.84 | 0.68,1.04 | 0.93 | 0.70,1.22 | 0.94 | 0.70,1.26 | 1.06 | 0.71,1.58 |
| CMV >4.9 log10 copies/ml | 108 | *17* | 50 | *18* | 173 | *20* | 0.78 | 0.59,1.02 | 0.78 | 0.54,1.11 | 0.71 | 0.50,1.0 | 0.60 | 0.37,0.97 |
| Coronavirus OC43 | 13 | *2* | 6 | *2* | 33 | *4* | 0.52 | 0.27,0.99 | 0.55 | 0.23,1.33 | 0.84 | 0.41,1.73 | 1.16 | 0.43,3.14 |
| Coronavirus NL63 | 8 | *1* | 3 | *1* | 23 | *3* | 0.48 | 0.21,1.08 | 0.38 | 0.11,1.29 | 0.41 | 0.15,1.08 | 0.43 | 0.11,1.69 |
| Coronavirus HKU | 4 | *0.6* | 1 | *0.4* | 20 | *2* | 0.27 | 0.09,0.80 | 0.16 | 0.02,1.17 | 0.37 | 0.1,1.17 | 0.24 | 0.03,2.0 |
| Coronavirus 229e | 1 | *0.2* | 1 | *0.4* | 6 | *0.7* | 0.21 | 0.02,1.72 | 0.46 | 0.06,3.90 | 0.45 | 0.05,3.92 | 1.18 | 0.13,10.55 |
| HBOV | 47 | *8* | 22 | *8* | 102 | *12* | 0.62 | 0.43,0.89 | 0.62 | 0.38,1.01 | 0.63 | 0.40,1.0 | 0.71 | 0.38,1.33 |
| HMPV A/B | 46 | *7* | 25 | *9* | 22 | *3* | 2.96 | 1.76,4.97 | 3.67 | 2.03,6.64 | 4.78 | 2.62,8.71 | 8.53 | 4.31,16.86 |
| Influenza A | 20 | *3* | 10 | *4* | 7 | *0.8* | 4.22 | 1.77,10.08 | 4.90 | 1.83,13.13 | 5.51 | 2.18,13.93 | 5.86 | 1.90,18.09 |
| Influenza B | 15 | *2* | 5 | *2* | 9 | *1* | 2.32 | 1.01,5.35 | 1.77 | 0.59,5.37 | 3.43 | 1.32,8.92 | 3.53 | 1.02,12.24 |
| Influenza C | 1 | *0.2* | 1 | *0.4* | 6 | *0.7* | 0.22 | 0.03,1.80 | 0.48 | 0.06,4.04 | 0.31 | 0.03,2.74 | 0.76 | 0.08,7.44 |
| Parainfluenza 1 | 11 | *2* | 6 | *2* | 1 | *0.1* | 16.02 | 2.06,124.71 | 19.93 | 2.38,167.10 | 23.4 | 2.84,192.5 | 37.9 | 4.02,356.6 |
| Parainfluenza 2 | 8 | *1* | 6 | *2* | 4 | *0.5* | 2.83 | 0.84,9.47 | 5.08 | 1.41,18.28 | 2.06 | 0.50,8.42 | 6.07 | 1.35,27.41 |
| Parainfluenza 3 | 28 | *5* | 14 | *5* | 24 | *3* | 1.64 | 0.94,2.86 | 1.80 | 0.92,3.54 | 2.37 | 1.27,4.44 | 2.53 | 1.11,5.75 |
| Parainfluenza 4 | 15 | *2* | 10 | *4* | 9 | *1* | 2.23 | 0.96,5.15 | 3.73 | 1.48,9.37 | 3.93 | 1.53,10.12 | 8.04 | 2.74,23.60 |
| Paraechovirus | 59 | *9* | 20 | *7* | 68 | *8* | 1.21 | 0.84,1.75 | 0.86 | 0.51,1.45 | 2.38 | 1.53,3.72 | 1.51 | 0.75,3.04 |
| Rhinovirus | 152 | *24* | 65 | *23* | 162 | *19* | 1.36 | 1.05,1.74 | 1.26 | 0.91,1.75 | 2.11 | 1.54,2.88 | 2.42 | 1.58,3.70 |
| RSV | 150 | *24* | 84 | *30* | 15 | *2* | 17.76 | 10.27,30.71 | 24.14 | 13.53,43.07 | 25.5 | 12.86,50.3 | 39.5 | 18.7,83.33 |

a. OR adjusted for age category

b. OR adjusted for age in months and all other pathogens

c. Four cases, none CXR+, had clinical signs or history of measles and were tested for measles by NP/OP PCR; two were positive.
