# Supplementary material for: The Etiology of Pneumonia in HIV-uninfected Children in Kilifi, Kenya: Findings From the Pneumonia Etiology Research for Child Health (PERCH) Study
Source: Pediatr Infect Dis J. 2021 Aug 25;40(9):S29–39. doi: 10.1097/INF.0000000000002653 (PMC8448399; doi:10.1097/INF.0000000000002653)
Supplement: Supplementary file 9 [file inf-40-s29-s009.docx]

Supplemental Digital Content 9: Estimated causes of pneumonia in PERCH cases, by CXR status, severity and age

|  | **Etiologic Fraction (95% CrI)** | | | | | | | | |
| --- | --- | --- | --- | --- | --- | --- | --- | --- | --- |
|  | **All HIV- Cases** | **CXR+/HIV- Cases** | **CXR Consolidation** | **CXR Other Infiltrate** | **CXR Normal** | **CXR+ Severe*** | **CXR+ Very Severe*** | **CXR+ Age < 1** | **CXR+ Age > 1** |
| **Aetiology** |  |  |  |  |  |  |  |  |  |
| **Bacteria** |  |  |  |  |  |  |  |  |  |
| B. pertussis | 0.1 (0, 0.5) | 0 (0, 0.4) | 0 (0, 0.8) | 0 (0, 0.6) | 0 (0, 0.4) | 0 (0, 0.6) | 0 (0, 0.9) | 0 (0, 0.6) | 0 (0, 0.8) |
| C. pneumoniae | 0 (0, 0.3) | 0 (0, 0.4) | 0 (0, 0.8) | 0.1 (0, 0.6) | 0.1 (0, 0.7) | 0 (0, 0.6) | 0.1 (0, 0.9) | 0 (0, 0.6) | 0.1 (0, 0.8) |
| Enterobacteriaceae^1^ | 0.2 (0, 1.3) | 0.3 (0, 2.8) | 0.3 (0, 2.5) | 0.2 (0, 1.9) | 0.2 (0, 2.1) | 0.3 (0, 3) | 0.3 (0, 3.5) | 0.3 (0, 2.6) | 0.4 (0, 4.8) |
| H. influenzae | 4.3 (1.4, 8) | 5.6 (1.8, 11.4) | 4.6 (0.8, 11.9) | 2.6 (0.6, 7.4) | 0.6 (0, 4.3) | 4.5 (0.6, 11.9) | 8.4 (1.8, 18.6) | 8.6 (2.6, 16.1) | 1.9 (0, 12.7) |
| Type b | 0.1 (0, 0.8) | 0.1 (0, 0.7) | 0.1 (0, 0.8) | 0 (0, 0.6) | 0.2 (0, 1.8) | 0.1 (0, 0.6) | 0.1 (0, 0.9) | 0.1 (0, 0.6) | 0.1 (0, 0.8) |
| Non-b | 4.2 (1.3, 7.8) | 5.5 (1.8, 11.4) | 4.5 (0.8, 11.9) | 2.6 (0.6, 7.4) | 0.4 (0, 3.9) | 4.4 (0.6, 11.9) | 8.4 (1.8, 18.6) | 8.5 (2.6, 15.5) | 1.8 (0, 12.7) |
| Legionella species | 0.1 (0, 1.3) | 0.3 (0, 2.8) | 0.2 (0, 2.5) | 0.1 (0, 1.2) | 0.3 (0, 3.2) | 0.3 (0, 3.6) | 0.3 (0, 3.5) | 0.2 (0, 1.9) | 0.5 (0, 5.6) |
| M. catarrhalis | 0.7 (0, 6.9) | 0.8 (0, 5.7) | 0.4 (0, 4.2) | 0.3 (0, 3.1) | 1.4 (0, 12.4) | 0.7 (0, 7.7) | 0.6 (0, 6.2) | 0.6 (0, 5.8) | 1 (0, 11.1) |
| M. pneumoniae | 0.2 (0, 0.8) | 0.3 (0, 1.4) | 1.1 (0, 3.4) | 0 (0, 0.6) | 0.3 (0, 1.4) | 0.1 (0, 1.2) | 0.4 (0, 2.7) | 0.3 (0, 1.3) | 0.4 (0, 2.4) |
| M. tuberculosis | 1.5 (0.3, 3.8) | 1.9 (0.4, 6) | 0.2 (0, 2.5) | 2.3 (0.6, 7.4) | 2.8 (0.4, 9.9) | 0.3 (0, 3) | 4.5 (0.9, 15) | 3.1 (0.6, 9.7) | 0.5 (0, 5.6) |
| N. meningitides | 0.1 (0, 1.1) | 0.3 (0, 2.8) | 0.3 (0, 3.4) | 0.1 (0, 1.9) | 0.3 (0, 3.2) | 0.3 (0, 3) | 0.3 (0, 3.5) | 0.2 (0, 2.6) | 0.4 (0, 4.8) |
| Non-fermenting gram-negative rods^2^ | 1.7 (0.2, 5.9) | 0.9 (0, 6.8) | 0.4 (0, 4.2) | 0.2 (0, 2.5) | 0.8 (0, 7.8) | 0.6 (0, 6) | 0.6 (0, 6.2) | 0.5 (0, 5.2) | 1.4 (0, 13.5) |
| Other streptococci and enterococci^3^ | 0.5 (0, 3.7) | 0.9 (0, 6) | 0.4 (0, 5.1) | 0.2 (0, 2.5) | 1.1 (0, 11) | 0.7 (0, 6) | 0.6 (0, 6.2) | 0.5 (0, 5.2) | 1.3 (0, 11.9) |
| S. aureus | 1.2 (0.2, 2.9) | 1.2 (0.4, 3.2) | 2.5 (0.8, 6.8) | 0.1 (0, 0.6) | 0.9 (0, 5.3) | 0.3 (0, 3) | 2 (0.9, 5.3) | 2 (0.6, 5.2) | 0.2 (0, 1.6) |
| S. pneumoniae | 2.7 (1.4, 4.5) | 5.2 (2.8, 8.5) | 9.9 (5.9, 15.3) | 1.3 (0.6, 3.1) | 0.2 (0, 1.8) | 1.8 (0.6, 4.2) | 13.7 (8, 21.2) | 3.4 (1.3, 7.1) | 7.5 (4, 13.5) |
| VT | 1.6 (0.8, 2.9) | 3.4 (1.8, 6) | 6.2 (3.4, 11) | 1.2 (0.6, 3.1) | 0.1 (0, 1.1) | 1.8 (0.6, 4.2) | 5 (1.8, 9.7) | 0.2 (0, 1.3) | 7.3 (4, 12.7) |
| NVT | 1.1 (0.3, 2.4) | 1.9 (0.7, 3.9) | 3.7 (1.7, 7.6) | 0.1 (0, 1.2) | 0.1 (0, 1.4) | 0 (0, 0.6) | 8.6 (3.5, 15) | 3.3 (1.3, 6.5) | 0.2 (0, 2.4) |
| Salmonella species | 0.5 (0, 2.2) | 0.5 (0, 2.8) | 0.5 (0, 4.2) | 0.1 (0, 0.6) | 0.2 (0, 1.8) | 0.6 (0, 4.2) | 0.1 (0, 1.8) | 0.1 (0, 1.3) | 1 (0, 6.3) |
| **Fungi** |  |  |  |  |  |  |  |  |  |
| Candida species | 0.4 (0, 3.5) | 0.9 (0, 6.8) | 0.4 (0, 4.2) | 0.2 (0, 2.5) | 1.1 (0, 9.9) | 0.7 (0, 7.1) | 0.6 (0, 6.2) | 0.5 (0, 5.2) | 1.4 (0, 13.5) |
| P. jirovecii | 0.6 (0, 1.8) | 2 (0, 4.3) | 2.1 (0, 5.1) | 2.1 (0, 4.9) | 0 (0, 0.4) | 1.2 (0, 4.2) | 1.9 (0, 5.3) | 3.1 (0, 7.1) | 0.6 (0, 2.4) |
| **Viruses** |  |  |  |  |  |  |  |  |  |
| Adenovirus | 1.1 (0, 4.3) | 0.6 (0, 3.2) | 0.2 (0, 1.7) | 1.1 (0, 6.2) | 4.7 (0, 10.3) | 0.9 (0, 6) | 0.4 (0, 3.5) | 0.4 (0, 3.2) | 0.9 (0, 6.3) |
| Bocavirus | 0.1 (0, 0.6) | 0.2 (0, 1.8) | 0.2 (0, 2.5) | 0.2 (0, 2.5) | 0.4 (0, 3.5) | 0.3 (0, 3) | 0.1 (0, 0.9) | 0.3 (0, 2.6) | 0.1 (0, 1.6) |
| CMV | 0.1 (0, 1.3) | 0.2 (0, 1.8) | 0.3 (0, 2.5) | 0.2 (0, 2.5) | 0.2 (0, 2.1) | 0.1 (0, 1.2) | 0.3 (0, 2.7) | 0.2 (0, 1.9) | 0.2 (0, 2.4) |
| Coronavirus | 0.1 (0, 0.6) | 0.2 (0, 1.8) | 0 (0, 0.8) | 1.5 (0, 5.6) | 0.2 (0, 1.8) | 0.1 (0, 0.6) | 0.3 (0, 2.7) | 0.1 (0, 1.3) | 0.4 (0, 3.2) |
| HMPV A/B | 7.5 (5.1, 10.4) | 10.5 (7.1, 14.6) | 10 (5.9, 14.4) | 11.2 (8, 15.4) | 5.7 (2.1, 9.2) | 10.2 (6.5, 15.5) | 11.1 (6.2, 17.7) | 13.1 (9.7, 18.7) | 7.3 (1.6, 12.7) |
| Influenza | 3.8 (1.6, 6.1) | 2.3 (0, 5.3) | 2.6 (0, 7.6) | 0.8 (0, 4.3) | 3.3 (0, 7.4) | 1.9 (0, 6) | 2.8 (0, 7.1) | 2.3 (0, 5.8) | 2.3 (0, 7.1) |
| A | 2.6 (0.8, 4.3) | 1.6 (0, 4.3) | 1.2 (0, 5.1) | 0.7 (0, 4.3) | 1.9 (0, 5) | 1.4 (0, 4.8) | 2 (0, 6.2) | 1.6 (0, 4.5) | 1.7 (0, 6.3) |
| B | 1.2 (0, 2.6) | 0.6 (0, 2.1) | 1.2 (0, 4.2) | 0.2 (0, 1.2) | 1.4 (0, 4.3) | 0.5 (0, 2.4) | 0.5 (0, 2.7) | 0.6 (0, 2.6) | 0.6 (0, 3.2) |
| C | 0 (0, 0.2) | 0.1 (0, 0.7) | 0.2 (0, 1.7) | 0 (0, 0.6) | 0 (0, 0.4) | 0 (0, 0.6) | 0.3 (0, 1.8) | 0.1 (0, 1.3) | 0 (0, 0.8) |
| Parainfluenza | 6.7 (4.5, 9.3) | 8.1 (3.6, 13.2) | 12.3 (5.9, 18.6) | 7.4 (2.5, 11.7) | 6.7 (2.8, 10.3) | 7.2 (1.2, 13.7) | 8.9 (4.4, 15.9) | 5.7 (0.6, 11) | 11 (4, 19.8) |
| 1 | 2.1 (1, 3.3) | 2.4 (0.7, 4.3) | 6.3 (3.4, 10.2) | 0 (0, 0.6) | 1.8 (0, 3.9) | 0.4 (0, 1.8) | 5.6 (3.5, 9.7) | 0.5 (0, 1.9) | 4.9 (1.6, 8.7) |
| 2 | 0.1 (0, 0.8) | 1.1 (0, 3.2) | 0.6 (0, 3.4) | 0.1 (0, 1.2) | 0 (0, 0.4) | 2.3 (0, 5.4) | 0 (0, 0.9) | 0 (0, 0.6) | 2.4 (0, 7.1) |
| 3 | 3.3 (1.9, 5.1) | 2.6 (0, 6) | 5.2 (0, 10.2) | 4 (0, 7.4) | 4.8 (1.4, 7.8) | 3.6 (0, 7.7) | 1.1 (0, 5.3) | 2.7 (0, 7.1) | 2.4 (0, 7.1) |
| 4 | 1.3 (0, 2.4) | 1.9 (0, 4.6) | 0.2 (0, 1.7) | 3.3 (0, 6.8) | 0.1 (0, 0.7) | 1 (0, 4.2) | 2.2 (0, 6.2) | 2.5 (0, 5.2) | 1.3 (0, 5.6) |
| PV/EV | 8 (4.8, 11.6) | 3.9 (0, 7.5) | 2.7 (0, 6.8) | 9.9 (6.2, 14.2) | 15.4 (11.3, 21.3) | 5.6 (0, 11.3) | 0.4 (0, 3.5) | 0.3 (0, 2.6) | 8.4 (0, 15.9) |
| Rhinovirus | 17.9 (12.4, 24.7) | 14 (8.5, 21) | 15.8 (8.5, 23.7) | 24.3 (18.5, 30.9) | 27.5 (20.6, 36.2) | 15.7 (7.1, 24.4) | 10.6 (0, 21.2) | 1.9 (0, 9.7) | 29 (18.3, 42.1) |
| RSV A/B | 28.2 (24.4, 33) | 37.1 (31.3, 44.1) | 32 (23.7, 40.7) | 32.8 (26.5, 39.5) | 18 (12.1, 24.1) | 44 (36.3, 54.8) | 29.7 (23, 38.9) | 51.8 (43.9, 61.3) | 19 (13.5, 26.2) |
| Not otherwise specified** | 11.6 (0, 20.3) | 1.7 (0, 10) | 0.5 (0, 5.1) | 0.4 (0, 3.7) | 7.6 (0, 25.5) | 1.5 (0, 13.1) | 1 (0, 9.7) | 0.7 (0, 7.1) | 2.8 (0, 21.4) |
| **Summary Estimates** |  |  |  |  |  |  |  |  |  |
| Bacteria*** | 12.3 (7.2, 21.1) | 16.4 (9.6, 25.6) | 20.7 (12.7, 30.5) | 5.3 (1.9, 11.7) | 6.5 (0.4, 20.2) | 10.3 (3, 22) | 27.5 (16.8, 40.7) | 16.6 (9, 26.5) | 16.1 (6.3, 33.3) |
| Viruses | 73.6 (65.4, 83.3) | 77.1 (67.3, 85.4) | 76.1 (65.3, 84.7) | 89.6 (82.1, 95.1) | 82 (68.4, 94.7) | 86 (72, 95.2) | 64.6 (50.4, 77) | 76 (65.8, 85.2) | 78.5 (60.3, 91.3) |

Abbreviations: CrI, credible interval, VT, vaccine-type, PV/EV, parechovirus/enterovirus; CMV, cytomegalovirus; RSV, respiratory syncytial virus.

* Severity assigned according to the 2005 World Health Organization case definition.

** Not otherwise specified represents pathogens not tested for.

*** The bacteria summary estimate excludes *M. tuberculosis*.

^1^ Enterobacteriaceae include: *E. coli*, Enterobacter species, and Klebsiella species, excluding mixed gram-negative rods.

^2^ Non-fermenting gram-negative rods include: Acinetobacter species and Pseudomonas species.

^3^ Other streptococci and enterococci include: *Streptococcus pyogenes* and *Enterococcus faecium*.
