# Supplementary material for: The Etiology of Pneumonia in HIV-uninfected Children in Kilifi, Kenya: Findings From the Pneumonia Etiology Research for Child Health (PERCH) Study
Source: Pediatr Infect Dis J. 2021 Aug 25;40(9):S29–39. doi: 10.1097/INF.0000000000002653 (PMC8448399; doi:10.1097/INF.0000000000002653)
Supplement: Supplementary file 10 [file inf-40-s29-s010.docx]

Supplemental Digital Content 10: Proportion of cases in whom Lower Respiratory Tract Infection is not identified as a primary or secondary discharge diagnosis

| ***Description^*^*** | ***All*** | | | ***Severe pneumonia*** | | | ***Very severe pneumonia*** | | |
| --- | --- | --- | --- | --- | --- | --- | --- | --- | --- |
|  | N | n | *%* | N | n | *%* | N | n | *%* |
| All PERCH cases | 634 | 177 | *28* | 310 | 42 | *14* | 324 | 135 | *42* |
| Cases with normal CXR | 286 | 113 | *40* | 114 | 19 | *17* | 172 | 94 | *55* |
| Cases with abnormal CXR | 284 | 42 | *15* | 168 | 19 | *11* | 116 | 23 | *20* |
| Consolidation +/-infiltrates | 121 | 23 | *19* | 65 | 11 | *17* | 56 | 12 | *21* |
| Infiltrates only | 163 | 19 | *12* | 103 | 8 | *8* | 60 | 11 | *18* |

* N refers to the total number of eligible cases in each category and n to the number of cases without a discharge diagnosis of Lower Respiratory Tract Infectio
