# Supplementary material for: The Etiology of Pneumonia in HIV-uninfected Children in Kilifi, Kenya: Findings From the Pneumonia Etiology Research for Child Health (PERCH) Study
Source: Pediatr Infect Dis J. 2021 Aug 25;40(9):S29–39. doi: 10.1097/INF.0000000000002653 (PMC8448399; doi:10.1097/INF.0000000000002653)
Supplement: Supplementary file 11 [file inf-40-s29-s011.docx]

**Supplemental Digital Content 11**

For more information about the sensitivity priors in the PERCH Integrated Etiology Analysis refer to [Appendix](https://www.thelancet.com/cms/10.1016/S0140-6736(19)30721-4/attachment/6303e6bf-f391-4045-b893-0a85966ff700/mmc1.pdf) Section III B 6 in [The PERCH Study Group. Causes of severe pneumonia requiring hospital admission in children without HIV infection from Africa and Asia : the PERCH multi-country case-control study. Lancet. 2019; 6736(19):1-23](https://www.thelancet.com/journals/lancet/article/PIIS0140-6736(19)30721-4/fulltext#section-7c530872-6235-4433-899c-b3f276970189).

**Table: Integrated etiology analysis input values for sensitivity and specificity of laboratory test measures**

|  |  | **Sensitivity Prior^a^** | |  |
| --- | --- | --- | --- | --- |
| **Specimen/test** | **Pathogen** | **Base** | **Reduced** | **Specificity** |
| Blood cultures^c^ | *-Streptococcus pneumoniae*  *-Haemophilus influenzae* | 5-20% | 1-13% | 100% |
|  | *-Moraxella catarrhalis*  *-Staphylococcus aureus*  -Nonfermentative gram-negative rods  -Candida species  -Non-pneumococcal streptococci, including enterococci | 5-15% | 1-10% |  |
|  | Salmonella species  Enterobacteriaceae  *Neisseria meningitidis* | 10-50% | 1-34% |  |
| NP/OP PCR | *-Streptococcus pneumoniae*  *-Haemophilus influenzae* | 50-90% | 15-55% | 1 − Control prevalence (ref SDC 8) |
|  | *-*Salmonella species  -Legionella species | 0.5-90% | 0.5-90% |  |
|  | -All other PCR targets | 50-90% | 50-90% |  |
| Whole blood PCR | *- Streptococcus pneumoniae* | 12-65% | 12-65% | 1 − Control prevalence (ref SDC 8) |
| Induced sputum | -*Mycobacterium tuberculosis* | 10-30% | 10-30% | 100% |

Abbreviation: SDC, Supplemental Digital Content.

a. Background information supporting choice of sensitivity priors provided in the all-site PERCH paper (The PERCH Study Group, 2019).

b. Base: > 1.5 mL blood culture volume (blood culture only) and no evidence of prior antibiotic exposure. Reduced < 1.5 mL or evidence of prior antibiotic exposure.

c. Direct evidence of the diagnostic sensitivity for *Streptococcus pneumoniae* and *Haemophilus influenzae* from vaccine probe studies. For all other pathogens we set the base blood culture sensitivity prior to 5-15%, with the exception of Salmonella species, Enterobacteriaceae and *Neisseria meningitidis*, for which we selected wider priors (10-50%) to reflect their greater uncertainty.
